# Taurine and Indicine Haplotype Representation in Advanced Generation Individuals From Three American Breeds

**DOI:** 10.3389/fgene.2021.758394

**Published:** 2021-10-18

**Authors:** Tamar E. Crum, Robert D. Schnabel, Jared E. Decker, Jeremy F. Taylor

**Affiliations:** ^1^ Division of Animal Sciences, University of Missouri, Columbia, MO, United States; ^2^ Informatics Institute, University of Missouri, Columbia, MO, United States

**Keywords:** indicine, taurine, hybrids, American breeds, selection, haplotype

## Abstract

Development of the American Breeds of beef cattle began in the 1920s as breeders and U. S. Experiment Station researchers began to create *Bos taurus taurus* × *Bos taurus indicus* hybrids using Brahman as the *B. t. indicus* source. By 1954, U.S. Breed Associations had been formed for Brangus (5/8 Angus × 3/8 Brahman), Beefmaster (½ Brahman × ¼ Shorthorn × ¼ Hereford), and Santa Gertrudis (5/8 Shorthorn × 3/8 Brahman). While these breeds were developed using mating designs expected to create base generation animals with the required genome contributions from progenitor breeds, each association has now registered advanced generation animals in which selection or drift may have caused the realized genome compositions to differ from initial expected proportions. The availability of high-density SNP genotypes for 9,161 Brangus, 3,762 Beefmaster, and 1,942 Santa Gertrudis animals allowed us to compare the realized genomic architectures of breed members to the base generation expectations. We used RFMix to estimate local ancestry and identify genomic regions in which the proportion of Brahman ancestry differed significantly from *a priori* expectations. For all three breeds, lower than expected levels of Brahman composition were found genome-wide, particularly in early-generation animals where we demonstrate that selection on beef production traits was likely responsible for the taurine enrichment. Using a proxy for generation number, we also contrasted the genomes of early- and advanced-generation animals and found that the indicine composition of the genome has increased with generation number likely due to selection on adaptive traits. Many of the most-highly differentiated genomic regions were breed specific, suggesting that differences in breeding objectives and selection intensities exist between the breeds. Global ancestry estimation is commonly performed in admixed animals to control for stratification in association studies. However, local ancestry estimation provides the opportunity to investigate the evolution of specific chromosomal segments and estimate haplotype effects on trait variation in admixed individuals. Investigating the genomic architecture of the American Breeds not only allows the estimation of indicine and taurine genome proportions genome-wide, but also the locations within the genome where either taurine or indicine alleles confer a selective advantage.

## Introduction

Indicine cattle were first imported into the United States from India in 1906 and then from Brazil in the 1920’s and were used via crossbreeding with taurine cattle and backcrossing to develop the *Bos taurus indicus* Brahman (Sanders, 1980) which has very little residual *Bos taurus taurus* within its genome (Chan et al., 2010). The American Breeds of beef cattle are populations that were developed in the United States beginning shortly after the introduction of the *B. t. indicus* cattle to capitalize on breed complementarity and heterosis for production and adaptation to heat stress and the nutritional limitations, parasites, and disease-causing pathogens prevalent in the southern tier of the country (Cartwright, 1970; Dickerson, 1970). Indicine × taurine crossbred individuals have been widely produced throughout subtropical and tropical regions of the world (Porto-Neto et al., 2014; Goszczynski et al., 2018) and the use of systematic crossbreeding programs world-wide has resulted in the development of at least 46 recognized indicine × taurine breeds (https://en.wikipedia.org/wiki/List_of_cattle_breeds). Breed Associations for the American Breeds began to be formed in the 1940’s and advanced generation composite animals now exist for the older Brangus, Beefmaster, and Santa Gertrudis breeds.

Brangus cattle were derived from animals created in public and private breeding experiments involving crosses between Angus (*B. t. taurus*) and Brahman cattle in Oklahoma, Mississippi, Texas, and Louisiana in the 1930’s and have been stabilized at an expected genome content of ⅜ Brahman and ⅝ Angus (http://afs.okstate.edu/breeds/cattle/brangus/index.html/). The American Brangus Breeders Association was formed in 1949 but was later renamed the International Brangus Breeders Association (https://gobrangus.com/jan-17-bj-con-lilley/). Santa Gertrudis cattle were initially developed on the King Ranch in Kingsville, Texas, where experimental crossbreeding between Shorthorn (*B. t. taurus*), and Brahman cattle between 1910 and 1920 led to the birth of the bull “Monkey” from which all registered Santa Gertrudis cattle descend (http://afs.okstate.edu/breeds/cattle/santagertrudis/index.html). However, the utilized Brahman bulls ranged in composition from ¾ to ⅞ *B. t. indicus* and, consequently, the Santa Gertrudis breed is considered to have a composition of ⅜ Brahman and ⅝ Shorthorn (Rhoad, 1949; Warwick, 1958). Santa Gertrudis was recognized as a breed by the United States Department of Agriculture in 1940. The foundation animals for the Beefmaster breed were developed beginning in 1908 as cross between Brahman, Shorthorn, and Hereford (*B. t. taurus*) on the Lasater Ranch in Falfurrias, Texas and are now maintained at an expected pedigree proportion of ½ Brahman, ¼ Hereford, and ¼ Shorthorn (Warwick, 1958). Beefmaster was recognized as a beef breed by the United States Department of Agriculture in 1954. These American Breeds of cattle now provide an interesting opportunity to study the genomic architectures of advanced generation composites with *a priori* known expected genomic breed proportions based on pedigree that have been exposed to natural selection for adaptation and artificial selection for beef performance traits.

Several approaches have been developed for the estimation of local ancestry (breed of origin of the two alleles present at specific loci) in admixed individuals, however, these applications have primarily been focused on recently admixed populations. Individuals from admixed populations have chromosomes that comprise mosaics of chromosomal segments originating from each of the ancestral populations (Thornton and Bermejo, 2014). On the other hand, global ancestry estimates predict the relative proportions of the ancestral genomes present in an admixed individual, which is an average of the local ancestry estimates, and ignores information pertaining to the variability among locus-specific ancestries (Tang et al., 2005). Drift and strong selection can lead to regions of the genome with ancestries that differ significantly from breed expectation and examination of these regions may identify candidate genes that are under selection and suggest the nature of the selected phenotype. We estimated local ancestry for registered Brangus, Santa Gertrudis, and Beefmaster animals that had been genotyped with the BovineSNP50, or derivative assays, and examined the average ancestries at specific chromosomal locations to identify regions of the genome that differ from expected global proportions both within and across breeds. Using the total number of haplotypes detected in each animal’s genome as a proxy for its generation number, we also contrasted the genomes of early- and advanced-generation animals to ascertain those genomic regions which had been exposed to recurrent selection within each of the breeds.

## Materials and Methods

### Genotype Data

Genotype data were obtained for deidentified individuals from the International Brangus Breeders Association, Beefmaster Breeders United, and Santa Gertrudis Breeders International Breed Associations (American Breed Associations) (Table 1). These individuals had been genotyped using at least one of 8 commonly used assays including the GeneSeek (Lincoln, NE) BOVG50v1, GGP-90KT, GGP-HDV3, GGP-LDV3 and GGP-LDV4, the Illumina (San Diego, CA) BovineHD and BovineSNP50, and the Zoetis (Kalamazoo, MI) i50K. PLINK1.9 (Purcell et al., 2007) was used to filter variants and individuals. SNP positions were based on the ARS-UCD1.2 bovine reference genome assembly (Rosen et al., 2020). Non-autosomal variants were removed from the data. Variants and individuals with genotype call rates <0.90 were also removed. Genotypes were phased using Eagle 2.4 (Loh et al., 2016) with a reference panel of haplotypes for 9,937 individuals genotyped with the BovineHD (HD) assay. Phased haplotypes were then imputed to the SNP content represented in the union of the HD and GeneSeek GGP-F250 (F250) assays using Minimac3 (Das et al., 2016). The multi-breed reference set created by Rowan et al. (2019) was used for genotype imputation. The reference panel contained 2,719 animals that had been genotyped with both the F250 and the HD assays, 25,772 animals genotyped with only the F250, and 7,218 animals genotyped with only the HD assay. Following imputation, each sample had genotypes for 836,118 variants.

**TABLE 1:** Genotyped samples for the American Breeds.

Assay^a^	No. Brangus	No. Beefmaster	No. Santa Gertrudis
BOVG50v1	0	836	264
GGP-90KT	688	0	6
GGP-HDV3	1,003	1,199	0
GGP-LDV3	0	36	756
GGP-LDV4	5,597	304	897
BovineHD	982	0	23
BovineSNP50	1,174	65	0
Zoetis i50K	14	1,332	0
Total^b^	9,458	3,772	1,946
Total^c^	9,161	3,762	1,942

a Assays used to genotype the samples. Genotypes were imputed to 836,118 variants.

b Number of individuals passing quality control after imputation and phasing.

c Number of individuals passing filtering for breed composition.

### Reference Panels

Local ancestry estimation requires a reference panel of genotypes for representatives of each ancestral population. We developed two reference panels for each American Breed. The first panel was identical for all three breeds and comprised animals registered by the American Angus Association, American Hereford Association, American Shorthorn Association, and American Brahman Breeders Association for which CRUMBLER (Crum et al., 2019) breed composition estimates were ≥85% to the respective breed (Table 2). The second reference panel was created using only individuals from the respective ancestral breeds (ANCESTRAL reference) (Table 2). Since some ancestry estimation software is influenced by unequal reference panel sample sizes (Maples et al., 2013; Crum et al., 2019), the breed possessing the smallest number of available individuals determined the approximate random sample size for the remaining breed(s). Reference panel individuals had been genotyped using one of 8 commercially available assays including the GeneSeek BOVG50v1, GGP-F250, GGP-HDV3, and GGP-LDV3, the BovineHD, BovineSNP50, i50K, and Irish Cattle Breeding Federation (Cork, Ireland) IDBv3, and were phased and imputed following the same procedures as for the American Breed individuals.

**TABLE 2:** Genotype data for registered individuals from 4 breeds used to generate reference panels.

Breed	No. Registered^a^	No. Individuals >85%^b^	CRUMBLER Reference Panel^c^	ANCESTRAL Reference: Brangus^d^	ANCESTRAL Reference: Santa Gertrudis^d^	ANCESTRAL Reference: Beefmaster^d^
Angus	6,699	252	200	997^e^	—	-
Hereford	3,651	227	200	—	—	500
Shorthorn	487	183	183	—	487	487
Brahman	954	361	200	954	500	500
Total	11,791	1,422	783	1,954	987	1,487

a Total number of available registered animals with genotypes.

b Number of registered animals identified with ≥85% CRUMBLER assignment probability to the respective breed.

c Random sample of ≤200 animals/breed from individuals with ≥85% CRUMBLER assignment probability to their respective breed.

d Ancestral breed sample sizes were determined by the breed with the fewest available registered animals.

e A random sample of 1,000 Angus resulted in 997 animals remaining following quality control for imputation and phasing.

### Local Ancestry Estimation

Gobena et al. (2018) used ADMIXTURE to estimate ancestry in an Angus × Brahman population, however, we have previously found the software to be sensitive to the order of animals within the input files (Crum et al., 2019) and consequently we used RFMix v2.03, for local ancestry and admixture estimation (Maples et al., 2013). RFMix partitions chromosomes into non-overlapping windows and infers ancestry for each window. If contiguous windows are assigned the same ancestry, RFMix concatenates the windows resulting in a variable number of haplotypes of varying length predicted for each individual. We first inferred local ancestry with a window size of 100 SNPs (spanning an average of ∼300 kb) using two reference panels for comparison. We next examined a window size of 25 SNPs (spanning on average ∼75 kb) for the ANCESTRAL reference panel. RFMix allows the specification of the number of generations separating the query samples from the initial reference population admixture event. However, because pedigree and generation information were not provided by the American Breed Associations, we used a generation interval of 5 years and the dates of formation of each Breed Association to arrive at an estimate of a maximum of 16 generations, with an average of about 8 generations for these individuals. This reflects the fact that first generation American Breed animals are continuously being generated and registered by breeders using superior animals from the requisite foundation breeds to capture the benefits of on-going selection within the numerically larger foundational breeds.

Many of the American Breed Associations allow the registration of the purebred, F_1_, and back-cross animals used to create first generation registered animals to enable a complete pedigree based on consistent registration numbers within their herdbook. To determine if some of these animals may have been genotyped and provided by the Breed Associations, we removed samples assigned by CRUMBLER to be ≥ 50% Brahman or ≥ 90% Angus or Shorthorn ancestry from the Brangus and Santa Gertrudis data, respectively (Brangus, *n* = 297; Santa Gertrudis, *n* = 4). For the Beefmaster, we removed samples with ≥ 90% assignment to Shorthorn, Hereford, or Brahman to remove potential purebred founders and any samples with ≤ 5% assignment to any one of these breeds to remove potential F_1_ individuals (*n* = 10).

### Generation Proxy

Genotypes used in this study were provided by the American Breed Associations with each animal’s identity anonymized and without pedigree or generation information. Since selection operates each generation, the cumulative effects of selection on a composite genome should increase with generation number. To enable the stratification of the animals within each American Breed based on generation number, we utilized the RFMix output to compute the total number of taurine and indicine haplotypes present within the diploid autosomal genome (2*N* = 58) of each animal and the average length of all haplotypes within the diploid genome. Under the assumption of selective neutrality, the number of haplotypes within the genome should increase each generation due to recombination and correspondingly, the average length of the haplotypes should decrease each generation. Purebred and F_1_ animals have 58 haplotypes represented as full length chromosomes and assuming an average of one crossover per chromosome pair each meiosis, back-cross (Purebred × F_1_) animals have, on average, 87 haplotypes which average ¾ of the chromosome length and F_2_ (F_1_ × F_1_) animals have, on average, 116 haplotypes which average ½ of the chromosome length.

### Genomic Divergence From Breed Expectation

Within each of the American Breeds, for the *i*th window within the genome we tested the null hypothesis that Ho: $\theta_{i}=\;\theta$ against the alternate hypothesis Ha: $\theta_{i}\neq \;\theta$ using the Z-statistic:

$${\text{Z}}_{\text{i}}=\frac{p_{i}-\theta }{\sqrt{Var\left(\theta_{i}\right)}}$$

Here, $\theta_{i}$ is the breed Brahman proportion and $p_{i}$ is the sample Brahman proportion within the *i*th window. The parameter θ represents the American Breed’s genome average expected Brahman proportion under selective neutrality in the absence of drift and was set to θ = 0.375 for Brangus and Santa Gertrudis, and 0.5 for Beefmaster. $\text{Var}\left(\theta_{i}\right)$ is the variation in Brahman proportion across windows throughout the genome under selective neutrality and in the absence of drift. $\text{Var}\left(\theta_{i}\right)$ cannot be estimated from the sample unless the null hypothesis is true and so to obtain an estimate of this parameter for each breed composition, we conducted a simulation using 1,000 animals per generation with each animal genotyped at the 836,118 loci with ARS-UCD1.2 reference genome coordinates. The number of recombination events per chromosome was assumed to follow a Poisson distribution with mean ( λ ) determined by chromosome length × recombination rate (e.g., λ = 158.532931 Mb × 0.01 recombination events per Mb = 1.58532931 for chromosome 1). The location of recombination events within each chromosome was simulated by sampling from a uniform distribution. For the Brangus and Santa Gertrudis simulation, 1,000 first generation ⅜ × ⅝ genomes were simulated by first creating 1,000 F_1_ × Purebred crosses (¾ taurine × ¼ indicine) which were then randomly mated to independently sampled F_1_ individuals. The first-generation animals were then randomly mated to produce 1,000 s generation individuals and so on for 8 generations of random mating. Ten replicate simulations were performed and within each replicate, θ was estimated as the average Brahman proportion across all genotyped loci and $\sqrt{Var\left(\theta_{i}\right)}$ was estimated as the square root of the variance of the Brahman proportion across all loci in the 8th generation individuals. Estimates of θ $\left(\hat{\theta }\pm SD\left(\hat{\theta }\right);\;0.3722\pm 0.0013\right)$ and $\sqrt{Var\left(\theta_{i}\right)}$ $\left(\hat{\sqrt{\text{Var}(\theta_{i})}}\pm SD\left(\hat{\sqrt{\text{Var}(\theta_{i})}}\right);\;0.0106\pm 0.0002\right)$ were obtained by averaging estimates across replicates. The Beefmaster simulation was identical to that for the Brangus and Santa Gertrudis except the 1,000 first generation ½ × ¼ × ¼ genomes were simulated by randomly sampling and mating 1,000 F_1_ genomes (½ taurine × ½ indicine). Estimates of θ and $\sqrt{Var\left(\theta_{i}\right)}$ were 0.4976±0.0013 and 0.0111±0.0002 , respectively. These estimates of $\hat{\sqrt{Var\left(\theta_{i}\right)}}$ were used in place of $\sqrt{Var\left(\theta_{i}\right)}$ in the calculation of the Z-statistics for each of the American Breeds. Controlling for the error rate in multiple testing of windows within each breed was achieved using adjusted *p*-values proposed by Benjamini and Hochberg. (1995) at a false discovery rate (FDR) ≤ 0.001. Significant regions were queried for QTL reported in the CattleQTLdb (https://www.animalgenome.org/cgi-bin/QTLdb/BT/search; Hu et al., 2019).

### Genomic Divergence Between Early- and Advanced-Generations

Within each of the American Breeds, we sampled ∼10% of individuals with the smallest and largest number of haplotypes with their genomes to characterize early- and advanced-generation individuals, respectively. Sampling was such that all animals within a haplotype number class were included resulting in slightly unequal sample sizes. There were 918, 207, and 400 animals in the early- and 955, 213, and 423 Brangus, Santa Gertrudis, and Beefmaster animals in the advanced-generation groups, respectively.

For the *i*th window within the genome, we tested the null hypothesis that Ho: $\theta_{ei}\;=\;\theta_{ai}$ against the alternate hypothesis Ha: $\theta_{ei}\;\neq \;\theta_{ai}$ using the Z-statistic:

$${\text{Z}}_{\text{i}}=\frac{p_{ei}\;-\;p_{ai}}{\sqrt{p_{i}\left(1\;-\;p_{i}\right)\left(\frac{1}{n_{e}}\;+\;\frac{1}{n_{a}}\right)}}.$$

Here, $\theta_{ei}$ and $\theta_{ai}$ are the *i*th window Brahman proportions within the early- and advanced-generation individuals within the breed, which are estimated by the sample proportions $p_{ei}$ and $p_{ai}$ , respectively. The statistic $p_{i}=\frac{n_{e}p_{ei}+\;n_{a}p_{ai}}{n_{e}+n_{a}}$ is the pooled estimate of the Brahman proportion when the null hypothesis is true and $n_{e}\;$ and $n_{a}\;$ are the numbers of haplotypes within the early- and advanced-generation individuals (twice the sample sizes), respectively. Multiple testing error rate was again controlled using Benjamini and Hochberg. (1995) adjusted *p*-values at a FDR ≤ 0.001. Significant regions were queried for QTL reported in the CattleQTLdb. This test was also applied genome-wide to test for differences in the global Brahman genome composition of early- and advanced-generation individuals.

## Results

### Reference Panels

RFMix generates three output files for each analysis: 1) the most likely reference population assignments for each haplotype found in a window, 2) the marginal probabilities of each reference population being the ancestral population of origin for the haplotypes found in the window, and 3) global diploid ancestry estimates (Maples et al., 2013). We utilized the most likely assignment files to define the breed of origin of haplotypes found within each window and then computed the relative frequencies of haplotype origin from each representative breed in each of the windows.

Following the strategy employed by Browning et al. (2016), we created two reference panels to evaluate the effects of panel size on the estimation of local ancestry. We first selected the Brahman, Angus, Hereford, and Shorthorn animals present in the CRUMBLER (Crum et al., 2019) reference panel which had been extensively developed and evaluated for use in global ancestry estimation for breeds commonly found in North America. Alternatively, for each of the American Breeds, an ANCESTRAL reference panel was created from all available registered animals. To avoid potential issues caused by unequal sample sizes, we randomly down-sampled the larger populations to the sample size for the breed with the fewest available samples (Table 2). For each of the American Breeds, the ANCESTRAL reference panel contained at least twice the number of animals from the ancestral populations than the CRUMBLER reference panel and ancestry estimates produced using the two panels were similar (Supplementary Figures S1–S3). However, the proportion of Hereford ancestry appeared to be underestimated and the proportion of Shorthorn ancestry overestimated when the CRUMBLER reference panel was used for local ancestry estimation in Beefmaster (Supplementary Figure S3). Consequently, we decided to utilize the ANCESTRAL reference panels for all further analyses.

### Window Size

**FIGURE 1:**
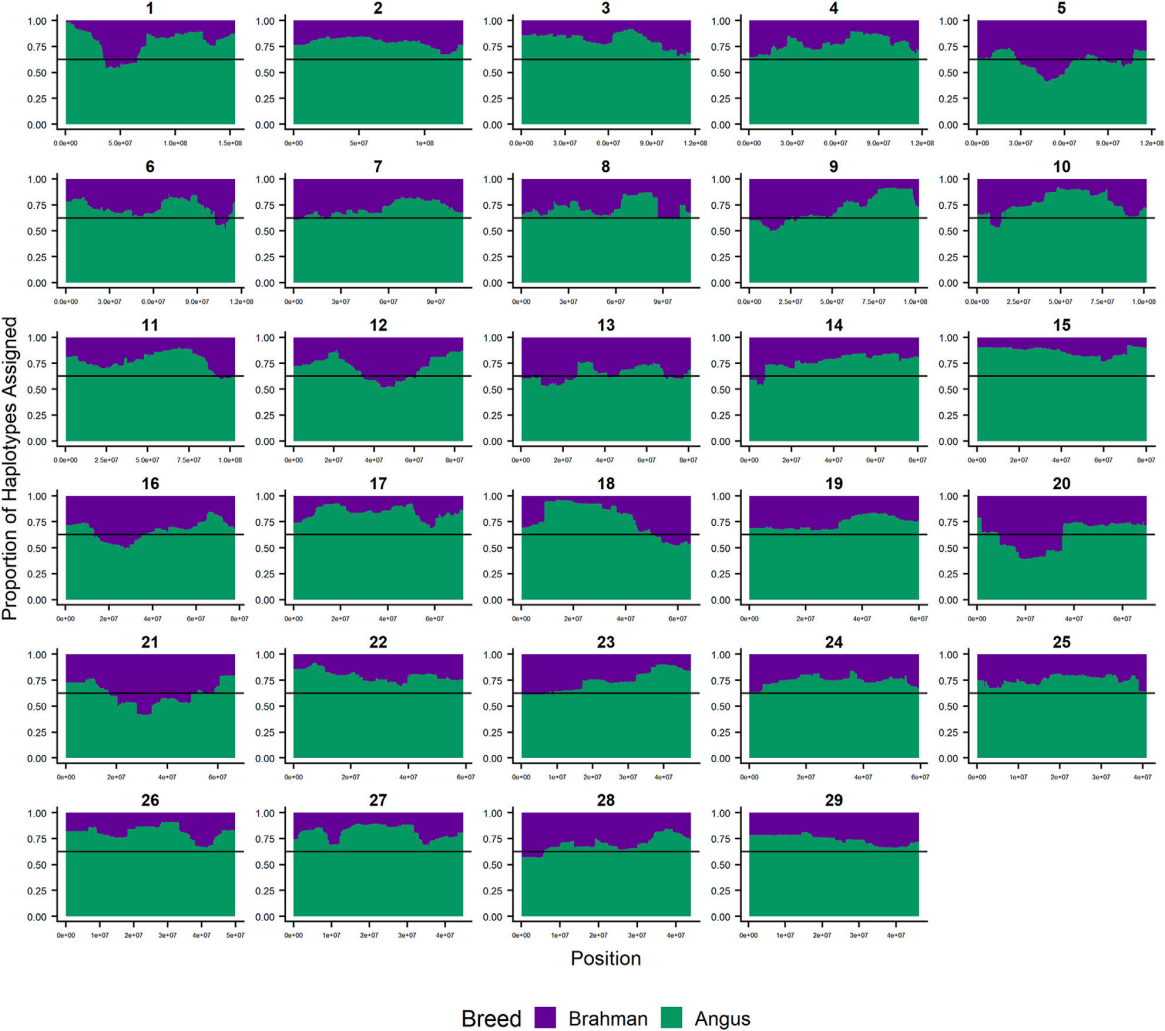
RFMix most likely assignment by chromosome for the ANCESTRAL reference panel for each 25 SNP window for Brangus. Brahman (purple) and Angus (green). Horizontal line indicates 5/8 expected Angus proportion.

**FIGURE 2:**
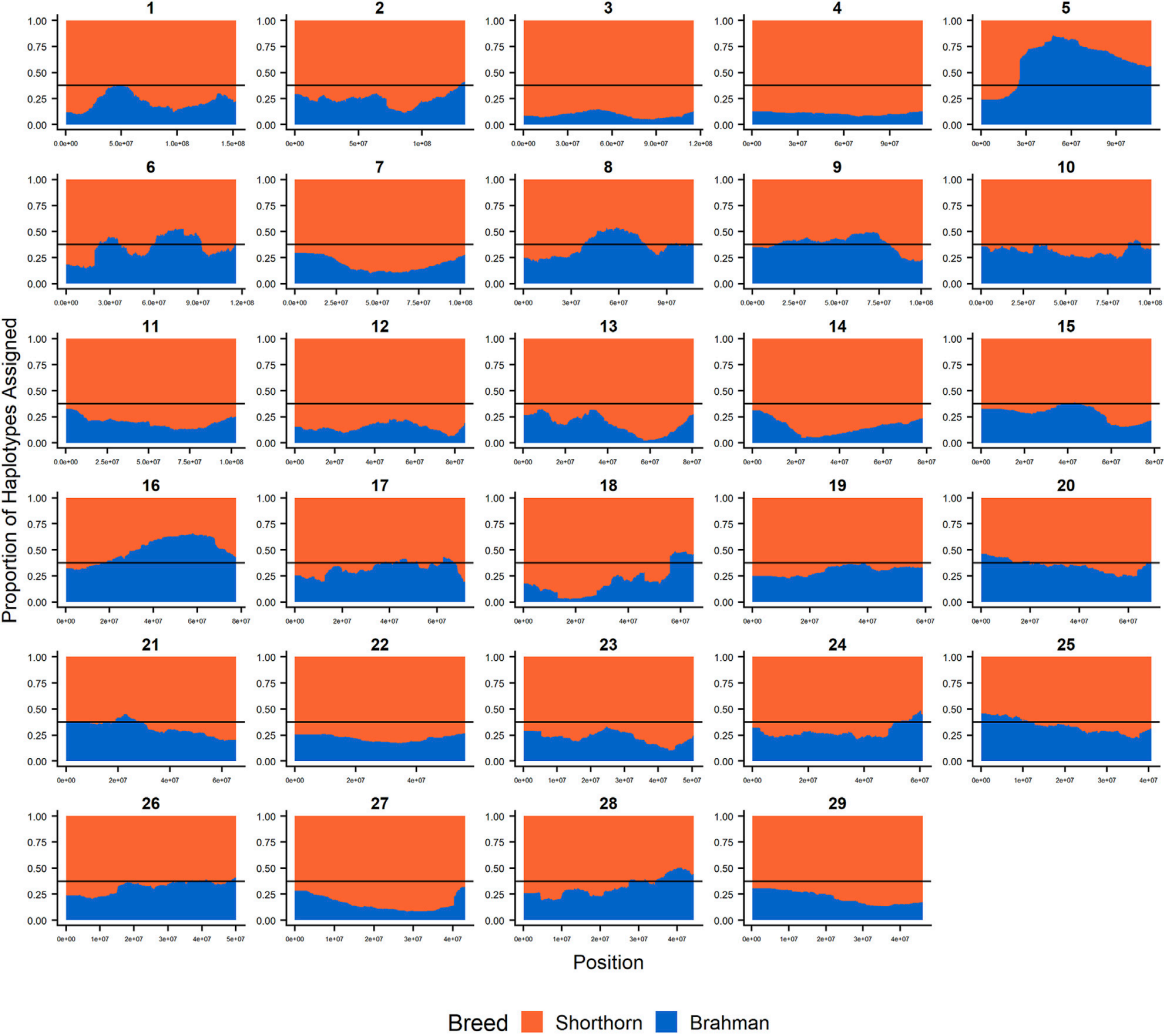
RFMix most likely assignment by chromosome for the ANCESTRAL reference panel for each 25 SNP window for Santa Gertrudis. Brahman (blue) and Shorthorn (orange). Horizontal line indicates 3/8 expected Brahman proportion.

**FIGURE 3:**
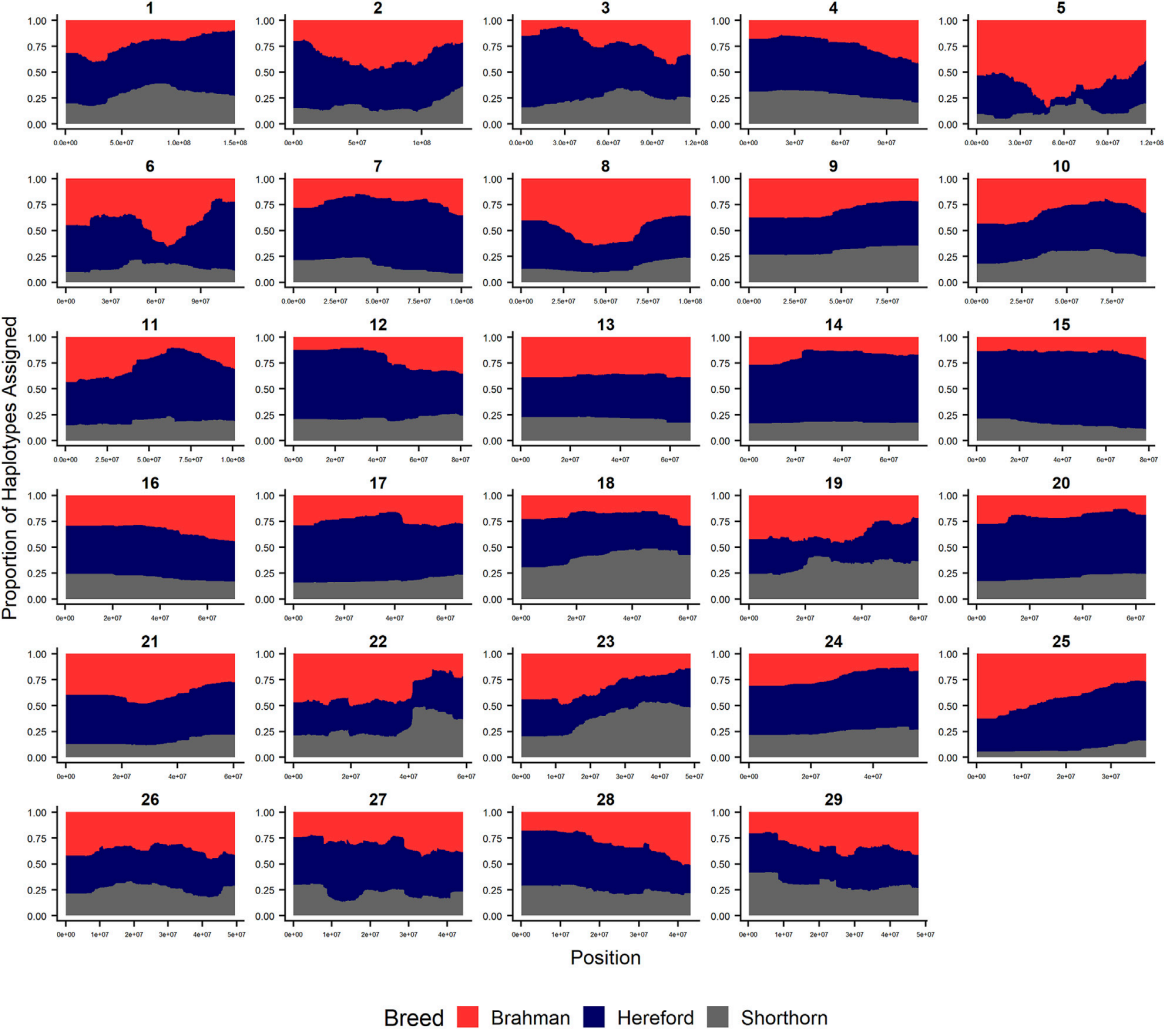
RFMix most likely assignment by chromosome for the ANCESTRAL reference panel for each 25 SNP window for Beefmaster. Brahman (red), Hereford (navy) and Shorthorn (gray). Beefmaster is expected to be ½ Brahman, ¼ Hereford and ¼ Shorthorn.

Since RFMix concatenates contiguous windows with the same ancestry to form extended haplotypes within individuals and considering the large number of SNPs used in this study, we were interested in whether a window size of 25 SNPs would impact the analysis in terms of resolution near recombination breakpoints or the ability to discriminate between haplotype breed of origin. Figures 1–3 show the local ancestry by chromosomal location for each of the American Breeds and are strikingly similar to the results shown in Supplementary Figures S1B, S2B, and S3B. Consequently, we conducted all further analyses using a window size of 25 SNPs.

### Generation Proxy

Using the RFMix output for all samples provided by the American Breed Associations (Table 2), we estimated the number of Angus, Shorthorn, Hereford, and Brahman haplotypes present within the genotyped individuals, and also the average length of these haplotypes by breed of origin. Haplotype metrics for all three of the American Breeds are presented in Supplementary Table S1 and suggest that the genomic architectures of the Brangus and Santa Gertrudis breeds are quite similar. Each breed possesses, on average, 50% more taurine than indicine haplotypes and the taurine haplotypes are almost twice the length of the indicine haplotypes.

The correlation between the number of Angus and Brahman haplotypes in each Brangus individual was 0.80, while the correlation between the number of Shorthorn and Brahman haplotypes in each Santa Gertrudis individual was 0.73. This follows our expectation that the total number of haplotypes from each of the foundation breeds increases in advanced generation animals due to recombination. However, the correlation between the number of Brahman and taurine (Shorthorn and Hereford) haplotypes in each Beefmaster individual was only 0.40. The correlation between the number of Brahman and Hereford haplotypes was 0.50 while the correlation between the number of Brahman and Shorthorn haplotypes was −0.11 suggesting that other forces such as selection may be influencing the evolution of the Beefmaster genome.

### Breed Ancestry

The average Brahman genome content was 25.81 ± 8.01% (±standard deviation among sampled individuals) for Brangus and 27.60 ± 7.05% for Santa Gertrudis (Table 3), far less than the 37.5% based upon breed expectations. Likewise, the Beefmaster had considerably fewer haplotypes of Brahman origin of smaller than average length (Supplementary Table S1) resulting in an average Brahman genome content of 30.84 ± 7.48%, far less than the breed expectation of 50%. The average Shorthorn genome content in Beefmaster individuals was 22.93 ± 8.01% and the average Hereford genome content was 46.23 ± 6.00%.

**TABLE 3:** Average ancestry to reference populations by chromosome for each of the American breeds^a^.

Chr	Brangus Av. Brahman % (SD)	Santa Gertrudis Av. Brahman % (SD)	Beefmaster Av. Brahman % (SD)	Beefmaster Av. Hereford % (SD)
1	21.69 (12.25)	23.03 (7.83)	22.51 (9.03)	48.00 (6.42)
2	21.07 (5.03)	24.70 (6.18)	34.79 (9.10)	47.57 (7.25)
3	19.18 (6.94)	***9.51 (2.83)***	22.67 (10.97)	52.48 (13.60)
4	22.06 (6.91)	***10.45 (1.21)***	24.74 (8.59)	47.69 (4.92)
5	**39.32 (8.54)^b^**	**63.28 (16.19)**	**60.85 (9.93)**	26.73 (11.78)
6	28.33 (7.86)	34.63 (10.73)	40.97 (12.79)	44.45 (14.61)
7	28.56 (6.36)	18.33 (6.26)	21.72 (4.82)	61.74 (5.08)
8	27.81 (7.90)	**36.88 (10.00)**	**51.00 (10.14)**	34.81 (6.59)
9	27.62 (13.19)	38.45 (8.42)	29.20 (6.34)	39.57 (2.97)
10	24.62 (10.82)	31.65 (4.43)	31.19 (8.18)	42.67 (4.29)
11	23.00 (8.83)	19.78 (4.82)	26.72 (10.87)	55.11 (9.10)
12	27.82 (10.95)	***14.46 (4.22)***	22.43 (9.65)	55.76 (11.09)
13	35.18 (6.93)	19.16 (8.68)	36.60 (1.17)	42.06 (1.56)
14	23.43 (7.82)	16.28 (6.98)	***17.20 (4.40)***	65.29 (3.95)
15	***13.78 (4.15)* ^c^**	28.19 (7.46)	***14.62 (2.11)***	70.17 (2.00)
16	33.27 (9.25)	**51.21 (11.00)**	34.93 (5.57)	45.16 (3.30)
17	***16.03 (5.90)***	32.78 (6.45)	24.40 (4.24)	57.11 (5.98)
18	25.00 (15.38)	21.66 (13.73)	***18.93 (3.75)***	38.68 (5.51)
19	26.11 (6.10)	30.69 (4.41)	36.49 (7.79)	29.70 (7.87)
20	**38.38 (13.24)**	34.82 (5.83)	19.22 (3.37)	59.62 (1.90)
21	**38.80 (10.37)**	31.52 (7.06)	38.74 (7.03)	45.54 (3.38)
22	20.87 (5.11)	21.67 (2.97)	37.35 (12.35)	32.77 (4.53)
23	24.40 (8.75)	22.80 (5.72)	31.03 (10.94)	29.10 (4.16)
24	24.80 (3.89)	28.83 (6.86)	21.05 (6.25)	53.63 (3.73)
25	24.87 (4.66)	33.40 (6.82)	**41.11 (10.78)**	50.42 (7.89)
26	19.11 (6.62)	32.93 (5.63)	36.43 (4.43)	37.57 (3.80)
27	***18.06 (6.56)***	14.82 (6.16)	31.44 (5.67)	47.51 (4.95)
28	28.82 (7.27)	34.05 (9.75)	32.30 (10.74)	43.56 (8.18)
29	26.63 (4.61)	20.33 (5.77)	33.86 (5.92)	36.25 (3.58)
Genome Wide	25.81 (8.01)	27.60 (7.05)	30.84 (7.48)	46.23 (6.00)

a RFMix local ancestry estimates averaged across all chromosome windows and throughout the genome. SD = standard deviation.

b Three chromosomes with largest Brahman content within each breed are indicated in bold.

c Three chromosomes with smallest Brahman content within each breed are indicated in bold and italics.

Table 3 shows that chromosome 5 consistently had the highest Brahman ancestry in all three breeds, but there was not a strong concordance between chromosomes with the highest or lowest Brahman ancestry across the breeds. Despite this, the correlations between estimated Brahman ancestry percentages across all 29 autosomes were 0.49 for Brangus and Santa Gertrudis, 0.43 between Brangus and Beefmaster, and 0.61 between Santa Gertrudis and Beefmaster which also share a Shorthorn ancestry. These correlations certainly indicate that, on a chromosomal basis, there is a tendency for the breeds to share elevated or reduced Brahman ancestry.

### Individual Ancestry

**FIGURE 4:**
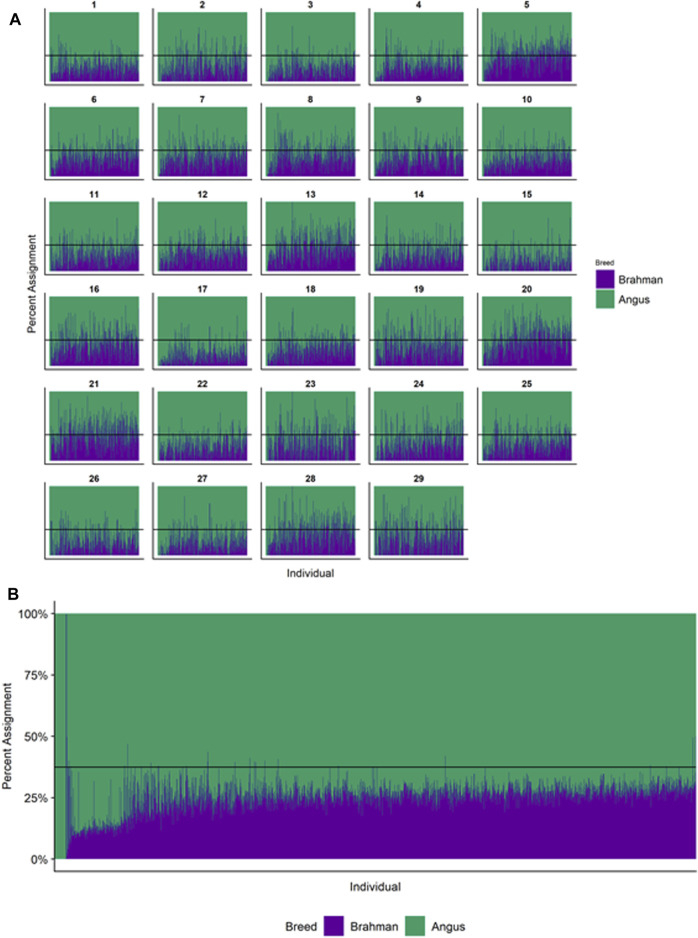
Ancestry estimated by RFMix for each Brangus individual plotted with animals sorted by total number of haplotypes. **(A)** Ancestry assignment for each chromosome, and **(B)** Genome-wide ancestry. Individuals are represented by vertical bars within each plot with Brahman proportion in purple and Angus proportion in green. Animals to the left on the *X*-axes have the fewest number of haplotypes predicted within their genomes. The black line represents the expected proportion of 3/8 Brahman.

**FIGURE 5:**
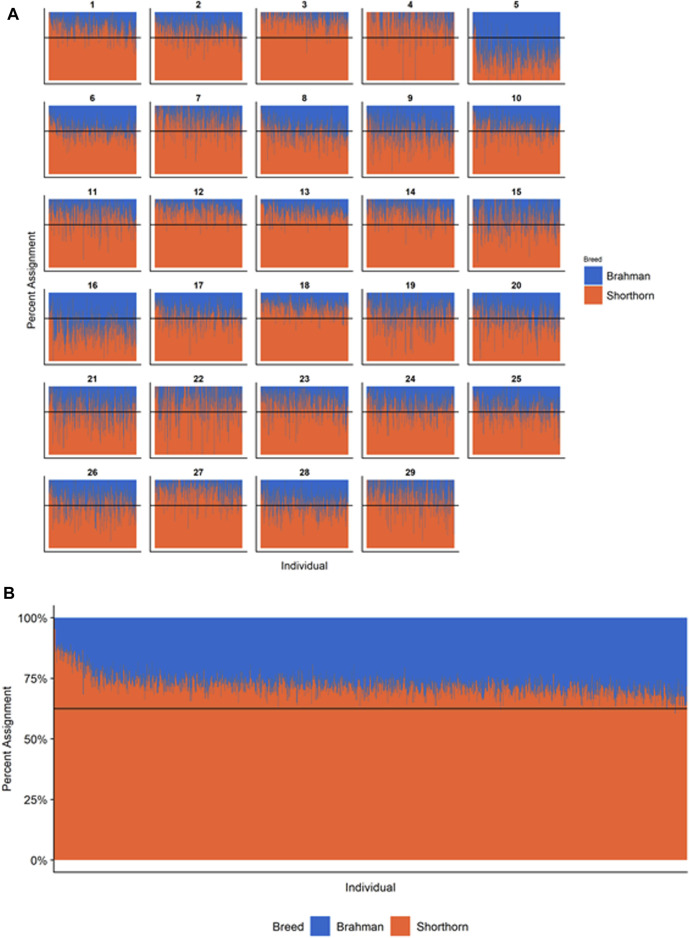
Ancestry estimated by RFMix for each Santa Gertrudis individual plotted with animals sorted by total number of haplotypes. **(A)** Ancestry assignment for each chromosome, and **(B)** Genome-wide ancestry. Individuals are represented by vertical bars within each plot with Brahman proportion in blue and Shorthorn proportion in orange. Animals to the left on the *X*-axes have the fewest number of haplotypes predicted within their genomes. The black line represents the expected proportion of 5/8 Shorthorn.

**FIGURE 6:**
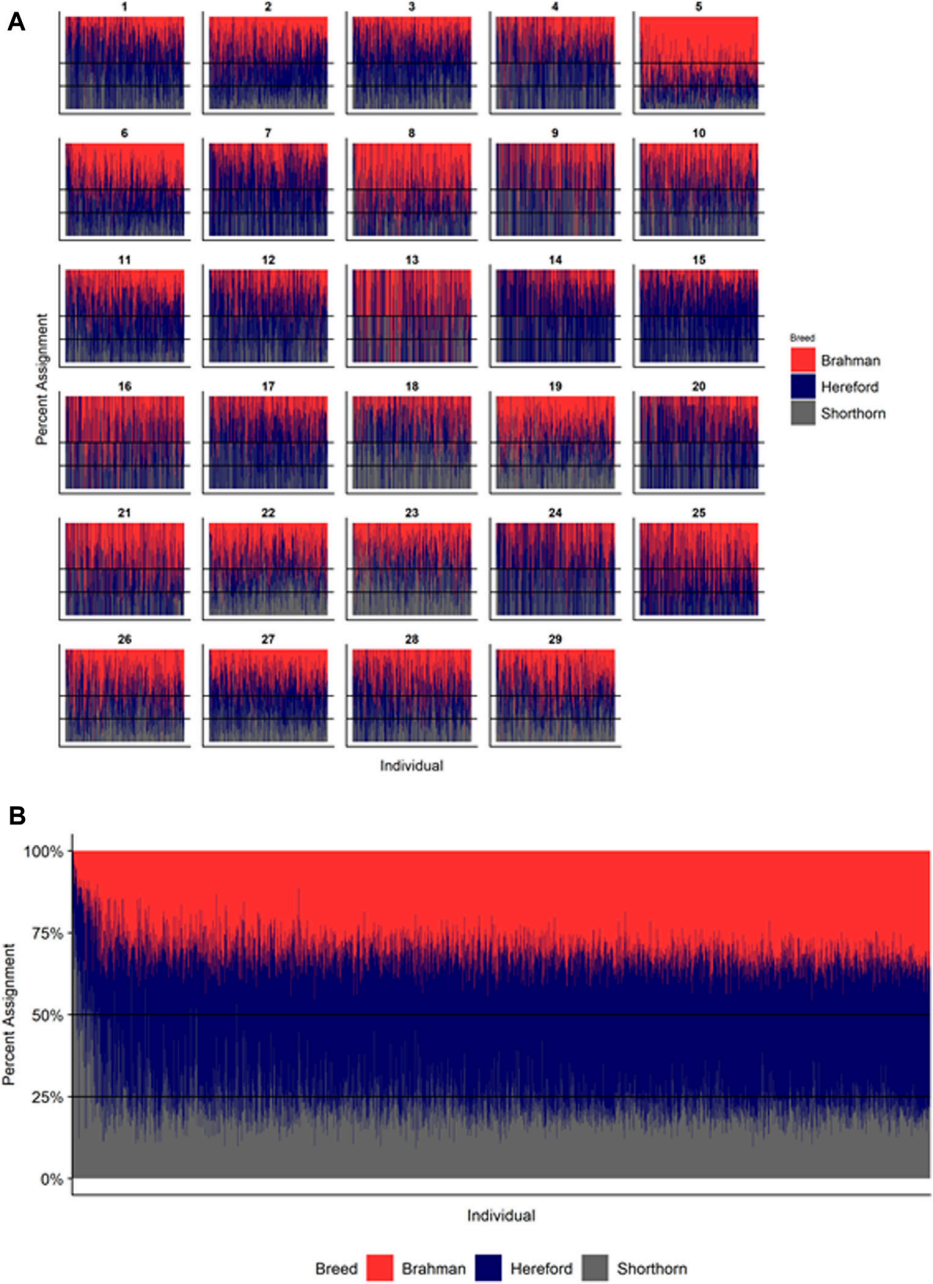
Ancestry estimated by RFMix for each Beefmaster individual plotted with animals sorted by total number of haplotypes. **(A)** Ancestry assignment for each chromosome, and **(B)** Genome-wide ancestry. Individuals are represented by vertical bars within each plot with Brahman proportion in red, Hereford in navy, and Shorthorn proportion in gray. Animals to the left on the *X*-axes have the fewest number of haplotypes predicted within their genomes.

Figures 4–6 show the proportions of ancestral breed inheritance for each individual by chromosome (panel A) and genome-wide (panel B). Individuals within each figure are sorted by the total number of haplotypes predicted within their genomes (*X*-axes and see Supplementary Table S1). For all three breeds, the proportion of Brahman content within the diploid genome increases with haplotype number, which we use as a proxy for the generation number of these individuals (see also Supplementary Figure S4). The results for individual chromosomes (Figures 4A, 5A, 6A) are not quite as obvious, however, the evolution of chromosome 5 clearly differs from most of the other chromosomes in all three breeds (see also Table 3).

### Genomic Divergence From Breed Expectation

**FIGURE 7:**
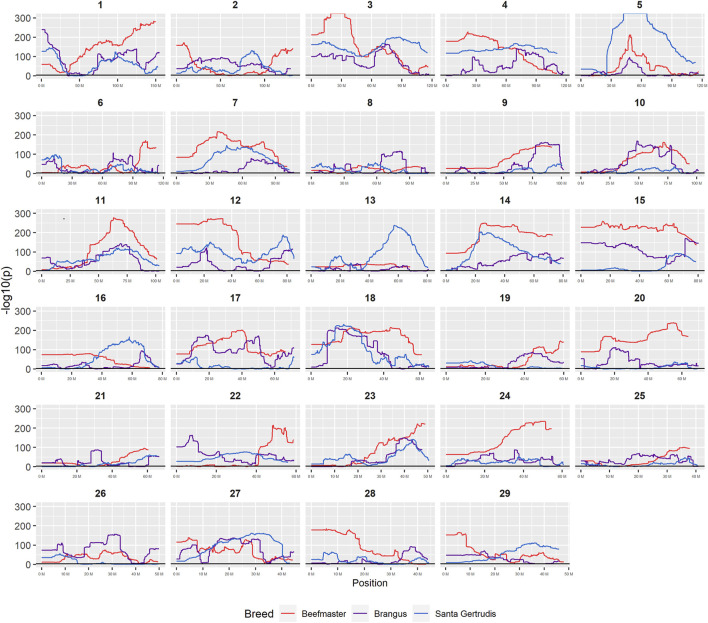
Plots of −log_10_P for tests of each genomic window for deviation from breed expected Brahman proportion by chromosome.

Nominal significance thresholds to achieve a FDR <0.001 using the Benjamini and Hochberg (1995) procedure were −log_10_ *p* = 3.0527 for Brangus, 3.0506 for Santa Gertrudis, and 3.0193 for Beefmaster. Using these values, 88.6% of tests performed for Brangus, 85.2% for Santa Gertrudis, and 95.2% for Beefmaster were significant. Values of the −log_10_P_i_ values for each American breed are plotted according to their chromosomal coordinates in Figure 7. These results reveal that most of the genomes of each of the American Breeds differ in Brahman composition from the expected breed proportion based upon pedigree when assuming selective neutrality and the absence of drift.

### Regions With the Greatest Brahman Divergence From Breed Expectation

The 5 most differentiated windows for Brahman genome content within each of the American breeds are in Table 4. These windows vary in size due to variation in the distribution of SNP locations throughout the genome, but also because RFMix concatenates contiguous windows where all individuals have the same ancestral population origin of haplotypes. Except for the locus on chromosome 5, all regions were enriched for taurine alleles. Table 5 contains beef trait QTL from the CattleQTLdb within the 1 Mb region centered on the regions enriched for taurine alleles in Table 4, except for the regions on chromosomes 1 and 18 in Brangus which are separately discussed.

**TABLE 4:** Five most significantly diverged regions from expected Brahman breed proportion by breed.

Chr	Start Pos.^a^ (bp)	Size^b^ (bp)	*P*_*i*_ ^c^	-log10(P)
**Brangus**				
1	376,390	4,082,465^d^	0.02	240.08
10	48,956,514	89,910	0.08	168.77
17	17,920,239	62,461	0.07	175.01
17	50,105,014	68,938	0.07	171.87
18	13,773,453	123,002	0.04	212.80
**Santa Gertrudis**				
3	86,676,910	332,543	0.05	201.02
5	42,298,343	184,428	0.79	>300^e^
13	57,508,582	82,009	0.02	237.25
14	23,570,574	96,661	0.05	204.54
18	17,876,786	67,551	0.03	232.08
**Beefmaster**				
1	146,960,035	92,662	0.10	283.59
3	19,467,876	57,666	0.08	>300^e^
11	63,215,699	125,829	0.10	277.62
12	32,131,527	236,418	0.10	273.19
15	14,921,941	334,777	0.12	259.04

a Window start coordinate.

b Window size in bp.

c Brahman proportion within the window.

d Contiguous windows concatenated by RFMix because full length haplotypes for all individuals were either Brahman or Angus.

e Due to rounding error *p* = 0.00 and -log_10_P = infinity.

**TABLE 5:** CattleQTLdb queries for QTL influencing traits selected in U. S. beef cattle for regions enriched for taurine alleles in American Breed cattle.

Chr	Region^a^ (bp)	Records^b^	Traits^c^	Most Likely QTL^d^	References
				**Brangus**	
10	49,001,469	50	28	Calving Ease; Stillbirth	Kühn et al. (2003); Schnabel et al. (2005); Seidenspinner et al. (2009)
				Age At Puberty	Hawken et al. (2012)
				Yearling Weight; Yearling Height	McClure et al. (2010); Snelling et al. (2010)
				Tick Resistance	Machado et al. (2010)
17	17,951,470	38	29	Birth Weight; Calving Ease; Stillbirth	Ashwell et al. (2005); McClure et al. (2010); Cole et al. (2011)
				Female Fertility	Cole et al. (2011)
				Average Daily Gain; Metabolic Body Weight; Slaughter Weight; Yearling Weight	MacNeil and Grosz (2002); Peters et al. (2012); Seabury et al. (2017); Akanno et al. (2018)
				Carcass Fat Thickness; Marbling	Casas et al. (2003); McClure et al. (2010)
				Bovine Respiratory Disease Susceptibility	Neupane et al. (2018)
17	50,139,483	40	11	Female Fertility	Gaddis et al. (2016)
				**Santa Gertrudis**	
3	86,843,182	41	17	Birth Weight; Calf Size; Calving Ease; Stillbirth	Sahana et al. (2011); Sugimoto et al. (2012)
				Female Fertility	Gaddis et al. (2016)
13	57,549,587	99	47	Birth Weight; Calf Size; Calving Ease; Gestation Length; Stillbirth	Kühn et al. (2003); Cole et al. (2011); Sahana et al. (2011); Sikora et al. (2011); Höglund et al. (2012); Saatchi et al. (2014b); Akanno et al. (2018)
				Male Fertility; Female Fertility; Age At Puberty; Estrus Interval	Sahana et al. (2010); Liu et al. (2017); Cole et al. (2011); Fortes et al. (2013); Höglund et al. (2015); Galliou et al. (2020)
				Stature; Yearling Weight	Kim et al. (2003); Cole et al. (2011)
				Carcass Yield Grade; Marbling	McClure et al. (2010); Saatchi et al. (2014b)
14	23,618,905	95	36	Birth Weight; Calving Ease; Gestation Length; Stillbirth	Kneeland et al. (2004); Maltecca et al. (2008); Snelling et al. (2010); Pausch et al. (2011); Martínez et al. (2014); Saatchi et al. (2014b); Michenet et al. (2016); Akanno et al. (2018); Smith et al. (2020)
				Male Fertility; Female Fertility; Twinning	Cobanoglu et al. (2005); Schnabel et al. (2005); Fortes et al. (2013); Hawken et al. (2012); Irano et al. (2016); Mota et al. (2017); Oliveira et al. (2017); Kiser et al. (2019); Galliou et al. (2020); Oliveira et al. (2020)
				Feed Intake; Feed Efficiency; Growth Rate; Stature; Weaning Weight; Yearling Weight; Carcass Weight; *Longissimus Dorsi* Muscle Area	Spelman et al. (1999); Kneeland et al. (2004); McClure et al. (2010); Snelling et al. (2010); Saatchi et al. (2014a); Saatchi et al. (2014b); Sharma et al. (2014); Brunes et al. (2021); Naserkheil et al. (2020); Smith et al. (2020); Srikanth et al. (2020)
				Carcass Fat Thickness; Marbling	Casas et al. (2003); McClure et al. (2010); Bolormaa et al. (2011); Abo-Ismail et al. (2018)
				Tick Resistance	Gasparin et al. (2007)
18	17,910,562	43	25	Calving Ease; Stillbirth	Kühn et al. (2003); Seidenspinner et al. (2009); Höglund et al. (2012)
				Metabolic Body Weight; Mature Weight; Carcass Weight; Feed Efficiency; Yield Grade; *Longissimus Dorsi* Muscle Area	Casas et al. (2003); Sherman et al. (2009); McClure et al. (2010); Saatchi et al. (2014b); Seabury et al. (2017); Brunes et al. (2021)
				Immune function; M. Paratuberculosis Susceptibility; Bovine Tuberculosis Susceptibility	Leach et al. (2010); Küpper et al. (2014); Wang et al. (2015); Richardson et al. (2016)
				**Beefmaster**	
1	147,006,366	180	41	Calving Ease; Stillbirth	Cole et al. (2011)
				Female Fertility; Estrus Interval	Cole et al. (2011); Melo et al. (2019)
				Growth Rate	Snelling et al. (2010); Seabury et al. (2017)
				Tick Resistance; Bovine Respiratory Disease Susceptibility	Mapholi et al. (2016); Sollero et al. (2017); Neupane et al. (2018)
3	19,496,709	33	19	Birth Weight; Gestation Length	Casas et al. (2003); McClure et al. (2010); Maltecca et al. (2011)
				Female Fertility	Cochran et al. (2013)
				Weaning Weight; Carcass Weight; Maturity Rate; Meat Yield	McClure et al. (2010); Doran et al. (2014); Saatchi et al. (2014b); Crispim et al. (2015)
				Carcass Fat Thickness	Casas et al. (2003)
				Bovine Tuberculosis Susceptibility	González-Ruiz et al. (2019)
11	63,278,614	74	45	Birth Weight; Calving Ease; Stillbirth	Cole et al. (2011); Sun et al. (2011)
				Male Fertility; Female Fertility; Estrus Interval	Höglund et al. (2009b); McClure et al. (2010); Kolbehdari et al. (2008)
				Body Size; Growth; Stature; Yearling Weight; Mature Weight; Carcass Weight	Snelling et al. (2010); McClure et al. (2010); Cole et al. (2011); Imumorin et al. (2011); Sun et al. (2011)
				Carcass Fatness; Marbling	Stone et al. (1999); McClure et al. (2010); Imumorin et al. (2011)
12	32,249,736	17	15	Calf Size; Calving Ease; Stillbirth	Sahana et al. (2011); Höglund et al. (2012)
				Age At Puberty	Stafuzza et al. (2019)
				Weaning Weight; Yearling Weight; Mature Weight; Carcass Weight; Mature Height	McClure et al. (2010); Saatchi et al. (2014b)
				Gastrointestinal Nematode Burden	Kim et al. (2013)
15	15,089,330	25	21	Birth Weight	McClure et al. (2010)
				Male Fertility	Druet et al. (2009); McClure et al. (2010)
				Growth; Yearling Weight; *Longissimus Dorsi* Muscle Area	McClure et al. (2010); Snelling et al. (2010); Li and Kim (2015); Li et al. (2017)
				Carcass Fatness	Casas et al. (2003); McClure et al. (2010)

a 1 Mb region centered on this coordinate.

b QTL/Association records returned from CattleQTLdb (August 10, 2021).

c Number of traits influenced by QTL/Associations.

d QTL for traits known to be selected in U. S. registered beef cattle for which taurine alleles are expected to have been selected.

### Genomic Divergence Between Early- and Advanced-Generations

The average number of haplotypes was 112.10 ± 9.03, 125.21 ± 11.16, and 103.60 ± 4.25 within the early-generation and 177.06 ± 5.12, 172.28 ± 5.13, and 140.71 ±  3.73 within the advanced-generation Brangus, Santa Gertrudis, and Beefmaster individuals, respectively. The average Brahman genome content was 15.92 ± 6.28, 21.39 ± 11.27, and 26.86 ± 14.44 within the early-generation and 29.44 ± 12.52, 32.22 ± 13.41, and 33.77 ± 1 3.90 within the advanced-generation Brangus, Santa Gertrudis, and Beefmaster individuals, respectively. The genome-wide Brahman proportions were significantly greater in the advanced-generation Brangus (*p* < 3.13E-23), Santa Gertrudis (*p* < 0.0002), and Beefmaster (*p* < 0.0012) individuals, respectively.

Nominal significance thresholds to achieve a FDR <0.001 using the Benjamini and Hochberg. (1995) procedure were −log_10_ *p* = 3.0317 for Brangus, 3.2030 for Santa Gertrudis, and 3.2399 for Beefmaster. Using these values, 91.23% of tests performed for Brangus, 62.61% for Santa Gertrudis, and 57.46% for Beefmaster were significant. The Brahman proportion was greater in the advanced-generation animals for all significant regions in Brangus and Santa Gertrudis and for 93.84% of the significant regions in Beefmaster. Values of the −log_10_P_i_ values for each American breed are plotted according to their chromosomal coordinates in Supplementary Figure S6. These results reveal that most of the genomes of advanced-generation individuals from each of the American Breeds have a greater Brahman composition than early-generation individuals.

### Regions With the Greatest Brahman Divergence Between Early- and Advanced-Generations

The 5 most differentiated windows for Brahman genome content between early and advanced-generation animals within each of the American Breeds are in Supplementary Table S2. For all 15 regions, the Brahman proportion was greater in the advanced-than in the early-generation animals. Supplementary Table S3 contains beef trait QTL from the CattleQTLdb within the 1 Mb region centered on the differentiated region in Supplementary Table S2.

## Discussion

### Reference Panels

Reference panel sample sizes have been shown to have significant effects on the accuracy of RFMix estimates (Maples et al., 2013). Reference panel sizes for analyses of local ancestry in human have ranged from as few as 19 samples to more than 500 samples per population (Maples et al., 2013; Browning et al., 2016) and human effective population size has been estimated to be 3,100 for Europeans and 7,500 for Yorubans (Tenesa et al., 2007). In cattle, the effective population size of most taurine breeds has been estimated to be about 100 (Bovine HapMap Consortium et al., 2009) and, consequently, we would expect that smaller reference panel sizes would be sufficient to capture the haplotypic diversity present within cattle breeds than for human. However, random samples of 200 individuals from each ancestral breed may not be sufficient to capture a large proportion of the haplotypic diversity within these breeds and larger sample sizes would certainly capture more of the rare haplotypes potentially leading to a greater accuracy of local ancestry estimation in admixed individuals (Maples et al., 2013). This is particularly important considering that the individuals in the reference panels and the founders of the American Breeds are separated by up to 16 generations and selection and drift may have caused large differences in haplotype frequencies between members of the groups. This appears to have impacted the estimation of local ancestry for the Beefmaster animals where proportions of Shorthorn and Hereford within the genome varied significantly depending on whether the CRUMBLER or ANCESTRAL reference panels were used. This may have been because the representation of horned Hereford and Line 1 Hereford animals was limited by sample size in the CRUMBLER reference panel and animals from these Hereford populations would have been prevalent at the time that the Beef master was initially formed.

### Generation Proxy, Breed, and Individual Ancestry

To examine genome evolution in these breeds, we utilized the total number of haplotypes present within the genomes of the animals as proxy for the generation number of the animal. Under selective neutrality we would expect the number of taurine and indicine haplotypes present within individuals to increase with generation number due to recombination among homologous chromosomes at each meiosis. Indeed, we found quite strong correlations (70–80%) between the numbers of indicine and taurine haplotypes within Brangus and Santa Gertrudis, but less so (50%) in Beefmaster. Strong selection for alleles found predominantly in one of the breeds will reduce the strength of this correlation and reduce the accuracy of the proxy for the prediction of generation number. However, we did not have any animals with known generation numbers with which to directly validate the utility of the proxy. Moreover, the results for Beefmaster may have been affected by our use of the most likely breed of haplotype origin, an absolute assignment, rather than the marginal probabilities of breed assignment. If the marginal probabilities of haplotype assignment to Shorthorn and Hereford are similar, but tend to favor one breed throughout the genome, we could see large differences in predicted Hereford and Shorthorn proportions genome-wide when, in fact, this is an artifact, and the true differences genome-wide are small. This would also artificially impact the correlations between taurine and indicine haplotype numbers in Beefmaster.

However, the results in Figures 4B, Figures 5B, Figures 6B are both encouraging and interesting. In all three figures, the animals with the fewest numbers of haplotypes within their genomes have by far the lowest Brahman genome content. We suspect that these animals are not early generation Brangus, Santa Gertrudis, or Beefmaster animals but are intermediary crosses (1/8 × 7/8 and ¼ × ¾) produced by cattle breeders generating first generation members of each of the breeds who decided to genotype these animals and provide them to their respective Breed Associations (see e.g., https://gobrangus.com/tperkins-breeding-up/). Despite this, the two interesting features of these plots are that the proportions of Brahman within all three of these breeds are far less than would be expected based upon the mating schemes used to produce the animals, and the proportion of Brahman within the genomes of advanced generation animals is consistently increasing supporting our results from the direct comparison of early- and advanced-generation animals. Our interpretation of these results are that breeders of American Breed cattle select very strongly for performance and type (coat color, polled, sheath score, performance) characteristics, particularly in early generation animals, resulting in a selective advantage for taurine alleles which creates widespread linkage drag throughout the genome dramatically reducing the Brahman content. However, in advanced-generation cattle, selection emphases change towards adaptive alleles (nutrient utilization, temperature tolerance, pathogen resistance) favoring indicine alleles. In the Beefmaster, the increase in Brahman genome content in advanced-generation animals appears to be primarily at the expense of the Shorthorn content (Supplementary Figure S5).

Goszczynski et al. (2018) used STRUCTURE to estimate the Brahman proportion within the genomes of 100 Argentinean registered Brangus animals to be 34.7%, which is very close to the breed expectation of 0.375 based upon pedigree. We suspect that the difference between this result and our finding reflects a very different selection history within the U. S. and Argentinian registered Brangus populations. Paim et al. (2020a) used ADMIXTURE applied to genotypes for 59 registered U.S Brangus cattle and estimated the Brahman proportion of their genomes to be 29.6%, on average, slightly larger than found in this study, but below breed expectation. Their estimates of breed proportion by chromosomal location show considerable similarity to our results in Figures 1–3, and they also found that chromosomes 5 and 15 had the lowest and highest Angus contents, respectively (Table 3), despite a much smaller sample size (Paim et al., 2020a).

### Genomic Divergence From Breed Expectation

To test genomic windows for divergence from breed expectations, we conducted a simulation to estimate the variance in the proportion of Brahman genome content that would be expected across loci in a large population of advanced generation individuals. The estimate of variance from the simulation was quite small $\left(\hat{\sqrt{Var(\theta_{i})}}\;\approx \;0.01\right)$ and because the overall Brahman genome content of individuals from each of the breeds was considerably smaller than that expected under neutrality, we found that very large proportions (>85%) of the genomes of each of the breeds were diverged from breed expectations. However, these values are very similar to the finding of Decker et al. (2012), who estimated that 82.41% of the genome of registered American Angus cattle was exposed to strong selection. The values of θ and $\text{Var}\left(\theta_{i}\right)$ used to calculate the Z-statistic are important for determining the number of performed tests that are deemed significant, however, the produced Z-statistics rank identically to the sample Brahman proportion estimates $p_{i}$ regardless of the values of θ and $\text{Var}\left(\theta_{i}\right)$ that are used. Our simulations are probably not representative of the generational mix within the samples we received from each Breed Association and the sample size simulated may not be representative of the number of animals used to generate each generation and it is possible that $\text{Var}\left(\theta_{i}\right)$ is considerably higher than the value we used. This would result in a smaller proportion of the genomes of each breed being found to have diverged from breed expectation. For example, if we had used $\hat{\sqrt{\text{Var}\left(\theta_{i}\right)}}$ = 0.02, 0.03, 0.04, or 0.05 (almost a 5-fold increase in standard deviation and 25 fold increase in variance) the statistical threshold for a FDR <0.001 would have been 3.16, 3.22, 3.32, and 3.45 in Brangus and we would have estimated that 69.5, 60.17, 47.3, or 35.6% of the Brangus genome was diverged from breed expectation, respectively. Whatever value of $\sqrt{\text{Var}\left(\theta_{i}\right)}$ is used, the most extreme test statistics are concordant with the most diverged genomic regions.

Figure 7 reveals that the patterns of divergence are complex but quite similar among the breeds for several chromosomes. The entirety of chromosome 25 possesses the least divergence, while chromosomes 1, 5, 11, 12, and 18 possess regions of very high divergence for all three breeds - characteristic of loci that have been strongly selected. Entire chromosomes are highly diverged for their Brahman composition including 15 in Brangus, 3 and 4 in Santa Gertrudis, and 14, 15, and 20 in Beefmaster. Chromosomes 8, 9, 13, 17, 20, 22, 24, and 29 reveal loci that have been exposed to selection in only a subset of the breeds. Selection for favorable alleles at multiple polygenes of large effect on a chromosome will generate a complex signature of selection that is dependent on initial phase relationships among the selected alleles and the relative intensities of selection applied to each of the alleles. Consequently, it is not likely to be a fruitful exercise to attempt to identity the underlying selected loci and phenotypes except perhaps for the largest effect loci and for loci that are well known to have been exposed to selection in these breeds.

Goszczynski et al. (2018) found a significant enrichment for Brahman alleles within the BoLA region of chromosome 23 (chr23:28,720,113–28,724,739), however, we found no evidence for this (Figure 7) and once again speculate that this reflects a different selection history for the U.S and Argentinian populations.

#### Coat Color and Polled Loci

##### Breed Characteristics

Brangus cattle are required to be polled (absence of horns) and to have solid black or red coat colors. Santa Gertrudis cattle may be horned or polled, with a light or dark solid red coat color preferred; roan (red coat with white patches) or white outside the underline or other spotting in other parts of the body disqualifies an animal from registration. Beefmaster cattle may be horned or polled and while brownish-red is the most common color, the breed has no color standards. Brahman cattle are primarily horned and have complex coat colors that can range from solid gray to brindled. Angus are polled due to the autosomal dominant *Celtic polled* allele, a complex structural insertion, at *Polled* located near the centromere of chromosome 1 (Brenneman et al., 1996; Medugorac et al., 2012) and have either solid black (American Angus Association) or red (American Red Angus Association) coat colors due to variation within *MC1R* located at 14,704,918–14,707,018 on chromosome 18. American Shorthorn can be horned or polled and can have red, white, or roan coat color patterns caused by variation at *KITLG* (Seitz et al., 1999) located at 18,245,986–18,317,616 on chromosome 5. American Hereford cattle can be horned or polled and have dark red to red-yellow coat colors. They are piebald with white on the head, ventral areas, lower legs, and tail switch which is inherited as an autosomal dominant due to structural variation located proximal to *KIT* at 70,157,944–70,262,786 Mb on chromosome 6 (Grosz and MacNeil, 1999; Whitacre, 2014). QTL associated with variation in white spotting in cattle have also been identified on chromosome 6 near *KIT* (Reinsch et al., 1999; Liu et al., 2009).

##### Selection Signatures at Coat Color and Polled Loci

Figure 7 confirms the selection of Angus alleles (Figure 1) at *Polled* and *MC1R* in Brangus (windows with strongest signal Chr1:376,390–4,458,855; $p_{i}$ = 0.02, -log_10_ *p* = 240.08; Chr18:13,773,453–13,896,455; $p_{i}$ = 0.04, −log_10_ *p* = 212.80) and Shorthorn alleles (Figure 2) at *MC1R* in Santa Gertrudis (Chr18:17,876,786–17,944,337; $p_{i}$ = 0.03, −log_10_ *p* = 232.08), respectively. These windows were among the 5 most differentiated windows for Brahman genome content in both breeds (Table 4). Paim et al. (2020b) found evidence of strong enrichment of Angus alleles at *Polled* in 59 Brangus animals, but not at *MC1R*. Ancestral breed frequency signatures on chromosome 5 are impacted by a very strongly selected locus enriched in frequency for Brahman alleles in all three of the American Breeds towards the center of the chromosome (Figures 1–3). Despite this, Figures 2, 3 show a reduced proportion of Shorthorn ancestry in Beefmaster, but an increased proportion of Shorthorn ancestry in Santa Gertrudis animals in the vicinity of *KITLG* on chromosome 5 presumably due to selection for the allele conferring red coat color and against that conferring white in Santa Gertrudis. Regions on chromosome 6 near *KIT* were strongly enriched for Angus alleles in Brangus (Chr6:70,550,407–70,635,750; $p_{i}$ = 0.14, −log_10_ *p* = 107.30), Brahman alleles in Santa Gertrudis (Chr6:71,137,845–71,233,574; $p_{i}$ = 0.51, −log_10_ *p* = 38.49), and Brahman alleles in Beefmaster (Chr6:68,154,476–68,192,608; $p_{i}$ = 0.66, −log_10_ *p* = 47.03). This suggests that selection has occurred for solid coat color patterns in all three breeds for both aesthetic and economic reasons due to losses caused by ocular squamous cell carcinoma, which is most common in Hereford animals that lack pigment around their eyes (Heeney and Valli, 1985).

#### Large Effect Locus Enriched for Ancestral Brahman Alleles

As the global proportion of indicine ancestry increases in *B. t. taurus* × *B. t. indicus* hybrids, coat color lightens, males tend to have more pendulous sheaths, body weight and condition scores increase, and tick and worm burdens decrease (Porto-Neto et al., 2014). *B. t. indicus* cattle tend to have a lower performance than *B. t. taurus* cattle under favorable conditions, but significantly outperform *B. t. taurus* cattle in extreme climates and environments where parasites, heat, and low inputs play important roles in the production system (Frisch and Vercoe, 1977; Frisch and Vercoe, 1984; Menjo et al., 2009; Porto-Neto et al., 2014). In almost all cases, the most significantly diverged regions are enriched for taurine alleles (Figures 1–3 and Figure 7). However, Brahman alleles are strongly enriched in frequency in all three of the American breeds in the central region of chromosome 5 (Figures 1–3). The window with the greatest Brahman composition was Chr5:48,183,014–48,275,554; $p_{i}$ = 0.59, −log_10_ *p* = 96.10 in Brangus, Chr5:42,298,343–42,482,771; $p_{i}$ = 0.79, −log_10_ *p* = >300 in Santa Gertrudis, and Chr5:48,090,162–48,159,989; $p_{i}$ = 0.84, −log_10_ *p* = 212.56) in Beefmaster. The window detected in Santa Gertrudis was almost 6 Mb from those found in Brangus and Beefmaster, but Figure 2 shows strong enrichment for Brahman alleles across the majority of chromosome 5 in Santa Gertrudis suggesting that selection may be acting on several loci on this chromosome in this breed. Querying the concatenated region Chr5:42,298,343–48,275,554 in the CattleQTLdb retrieved 458 QTL or association records for 87 different traits. Among these, loci responsible for variation in traits that are known to be under strong selection in beef cattle that have previously been reported in studies of indicine or indicine × taurine crossbred cattle include Birth Weight (Peters et al., 2012; Saatchi et al., 2014b), Male and Female Reproduction (Hawken et al., 2012; McDaneld et al., 2014; Buzanskas et al., 2017), Stature, Yearling Weight, Carcass Weight and *Longissimus Dorsi* Muscle Area (Imumorin et al., 2011; Pryce et al., 2011; Saatchi et al., 2014b), and intramuscular fatness measured as Marbling Score (Peters et al., 2012; Leal-Gutiérrez et al., 2019). Also within this region are QTL that have been found to be associated with Immune Function (Leach et al., 2010), Susceptibility to Bovine Respiratory disease (Neupane et al., 2018), and Tick Resistance (Gasparin et al., 2007).

The genes closest to the windows with the greatest Brahman ancestry in Brangus and Beefmaster are *HMGA2* (Chr5:47,819,475–47,966,336) and *MSRB3* (Chr5:48,330,041–48,511,868). In human, *HMGA2* has been associated with 62 traits (https://www.ebi.ac.uk/gwas/genes/HMGA2; Accessed Sept. 18, 2021), including body height, birth weight, systolic blood pressure, brain cortical volume, intelligence, and insomnia. Brahman females are known to suppress the birth weight of their calves (Dillon et al., 2015) to increase calving ease and reduce calf mortality. *MSRB3* has been associated with 33 traits in human (https://www.ebi.ac.uk/gwas/genes/MSRB3; Accessed Sept. 18, 2021) including brain cortical volume, snoring, lung function, height, and temperament. The window with the greatest Brahman ancestry in Santa Gertrudis overlaps *CPNE8*, associated with 14 human traits (https://www.ebi.ac.uk/gwas/genes/CPNE8; Accessed Sept. 18, 2021) including chronotype and circadian rhythm, IgG glycosylation, heart rate, and bipolar disorder. Consequently, this broad genomic region includes genes which may explain several of the physiological differences between *B. t. taurus* and *B. t. indicus cattle*, including birth weight, height, blood vessel morphology, hair morphology, disease resistance, and temperament.

#### Large Effect Loci Enriched for Ancestral Taurine Alleles

We queried CattleQTLdb for the remaining 12 loci in Table 4 to identify candidate QTL responsible for the enrichment of taurine alleles at these highly diverged loci and the results are in Table 5. We performed the query for 1 Mb regions centered on the window centers reported in Table 4 and restricted our attention to loci responsible for variation in traits known to be under artificial selection or potentially under natural selection in U. S. beef breeds and discovered in taurine or *B. t. taurus* × *B. t. indicus* hybrid populations. We also collapsed database entries from the same author with separate QTL identities into a single QTL when they co-localized within the genome. For example, the query for chromosome 14 region retrieved 5 database entries for Birth Weight from Snelling et al. (2010) with QTL identifiers 68615, 68626, 68630, 68658, and 68672 with coordinates Chr14:23,893,200–23,893,240, Chr14:23,946,416–23,946,456, Chr14:23,571,834–23,571,874, Chr14:23,853,791–23,853,831, and Chr14:23,421,913–23,421,953, respectively. These clearly all represent the same Birth Weight QTL and were collapsed into a single entry in Table 5. However, this feature of the CattleQTLdb makes it difficult to perform enrichment analyses, since the numbers of trait associations detected in any study is dependent upon the number of markers used. The CattleQTLdb contains 161,781 QTL from 1,049 publications representing 680 different traits (https://www.animalgenome.org/cgi-bin/QTLdb/index; Accessed August 11, 2021). The bovine autosomal genome length is 2,489.39 Mb in the ARS-UCD1.2 bovine reference genome assembly and so if QTL are randomly located through the genome, we would expect to find an average of 64.98 QTL within each 1 Mb window. We retrieved an average of 61.25 ± 45.49 QTL or Association entries for 27.83 ± 12.07 traits within the 12 genomic windows in Table 5 indicating that these genomic regions are typical for their QTL content.

Two features of Table 5 are particularly interesting. First, 7 of the 12 differentiated regions harbor single reports of QTL for Tick or Disease Resistance. However, the CattleQTLdb contains 2,923 Disease, 210 Parasite, 254 Immune Capacity, and 124 Parasite Pest Resistance QTL or Association entries, prior to coalescence to remove redundant QTL reporting. These data suggest that we should have found, on average, 1.41 QTL or Association entries within the CattleQTLdb per 1 Mb autosomal region. From the Poisson distribution, the probability of 1 or more CattleQTLdb entries per region is 0.76 and from the Binomial distribution, the probability of 7 or more regions possessing CattleQTLdb entries is 0.95. The second feature is that 11 of the 12 differentiated regions harbor QTL for Birth Weight or Calving Ease for which 1,180 and 4,105 QTL or Association entries are reported, respectively, in the CattleQTLdb. This corresponds to an average of 2.12 database entries per 1 Mb window and a probability of 0.57 of 11 or more of the windows having CattleQTLdb entries for Birth Weight or Calving Ease. However, the distributions of database entries for Birth Weight and Calving Ease by chromosome are not random, with 511 (43.3%) entries for Birth Weight on chromosome 6 and 1,968 (47.9%) entries for Calving Ease on chromosome 21. Removing these outlier chromosomes from the analysis leads to the expectation of 1.22 database entries per 1 Mb window and a probability of only 0.09 of finding entries for Birth Weight or Calving Ease in at least 11 of 12 randomly sampled 1 Mb windows.

Ten of the regions in Table 5 have CattleQTLdb entries for growth and weight traits and 7 of these are for Weaning Weight. We found 616 CattleQTLdb entries for Weaning Weight, again with an overrepresentation on chromosome 6 (146 or 24%). After removing chromosome 6 from the analysis, this corresponds to an average of only 0.20 database entries per 1 Mb window for the remainder of the autosomal genome and a probability of only 0.002 of finding at least 7 out of 12 randomly sampled 1 Mb genomic regions containing database entries for Weaning Weight. Four of the regions in Table 5 have CattleQTLdb entries for Marbling Score for which there are 441 CattleQTLdb entries corresponding to an average of only 0.18 database entries per 1 Mb window for the autosomal genome. This generates a probability of 0.12 of finding at least 4 out of 12 randomly sampled 1 Mb genomic regions containing database entries for Marbling Score. Finally, there are 19,572 entries in CattleQTLdb for Fertility indicating that we should have expected to find fertility trait QTL in all 12 of the genomic regions reported in Table 5.

These results suggest that artificial selection on production traits practiced by breeders of American Breed cattle is responsible for the surfeit of taurine alleles at the 12 loci in Table 5. Our analyses suggest that American Breed cattle breeders are placing the greatest selection emphases on growth, calving ease, and meat quality traits, which is consistent with the findings of Decker et al. (2012) in U.S registered Angus cattle.

### Genomic Divergence Between Early- and Advanced-Generations

Although statistical power was limited when comparing the Brahman content of the genomes of early- and advanced-generation animals due to the use of only ∼10% of the animals from each breed in each tail of the haplotype number distribution, these analyses clearly revealed that the Brahman proportion of the genome of these American breeds has increased with generation number and that this increase has occurred almost genome-wide.

There are several interesting features of Supplementary Table S3. First, we found two of the most differentiated 1 Mb regions between early- and advanced-generation American Breed animals to have no previously identified QTL within them. Considering an average of 64.98 QTL within each 1 Mb window for the CattleQTLdb, the probability of two such intervals in 15 randomly sampled 1 Mb genomic regions is small (*p* = 3.88E-55), indicating that the selected loci in these regions impact traits that have not been well studied in cattle, suggesting that they may have roles in environmental adaptation. Second, the locus on chromosome 6 at 71.5 Mb in Beefmaster supports our conjecture that Beefmaster breeders have selected for solid coat colors in advanced-generation animals, despite the fact that the breed does not have coat color standards. Finally, if we remove the locus on chromosome 6 at 71.5 Mb in Beefmaster, 11 of the 12 genomic regions with identified QTL harbor QTL that influence heat tolerance or animal health, immune response, parasite burden or disease susceptibility. The probability of 11 or all 12 regions harboring disease or parasite CattleQTLdb entries reduces to 0.18. These results suggest that these genomic regions have been selected to increase the proportion of Brahman alleles because they confer an adaptive advantage in American Breed cattle.

## Conclusion

The American breeds are advanced generation *B. t. taurus* × *B. t. indicus* hybrid cattle that were developed to capitalize on breed complementarity and heterosis to produce cattle that were suited to harsh, subtropical climates as well as disease and parasite threats, while still maintaining acceptable levels of growth and productivity. The breeds employed mating systems designed to produce cattle that were either ⅝ taurine and ⅜ indicine (Brangus and Santa Gertrudis) or ½ taurine and ½ indicine (Beefmaster). However, we found strong evidence that selection for polledness, coat color phenotypes, growth, calving ease, and intra-muscular fat content produced early-generation cattle with much smaller than expected indicine composition within the genomes of all three breeds. At least 85% of the genomes within each of the breeds possess less Brahman ancestry than expected in the absence of selection and drift. We utilized the total number of haplotypes predicted by RFMix in the diploid genome of each animal as a proxy for the generation number for each animal and when we ranked animals based on this proxy, we detected an increase in the Brahman genome content in advanced-generation cattle of all three breeds. By comparing the genomes of early- and advanced-generation animals within each breed, we found evidence for strong selection for indicine alleles which likely confer an adaptive advantage for heat tolerance and healthfulness in advance-generation animals.

## Data Availability

The datasets analyzed in this study belong to the respective Breed Associations. For access to the data, contact: Darrell Wilkes, Executive Vice President, International Brangus Breeders Association: dwilkes@gobrangus.com. Collin Osbourn, Executive Vice President, Beefmaster Breeders United: cosbourn@beefmasters.org, Webb Fields, Executive Director, Santa Gertrudis Breeders International: wfields@santagertrudis.com.
