# Supplementary material for: Taurine and Indicine Haplotype Representation in Advanced Generation Individuals From Three American Breeds
Source: Front Genet. 2021 Oct 18;12:758394. doi: 10.3389/fgene.2021.758394 (PMC8558500; doi:10.3389/fgene.2021.758394)
Supplement: Supplementary file 1 [file DataSheet1.docx]

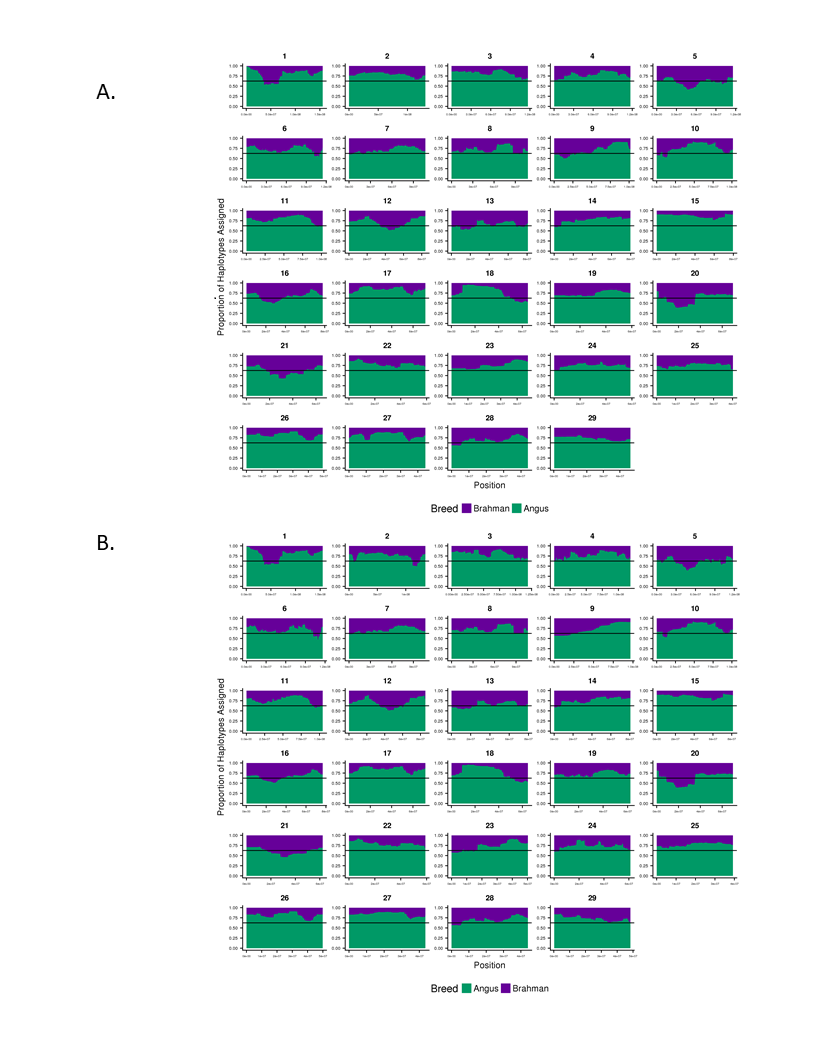


**Supplementary Figure S1.** RFMix most likely assignment by chromosome for the: A) CRUMBLER and B) ANCESTRAL reference panels. Brahman (purple) and Angus (green), for each 100 SNP window for Brangus. Horizontal line indicates 5/8 expected Angus proportion.

**
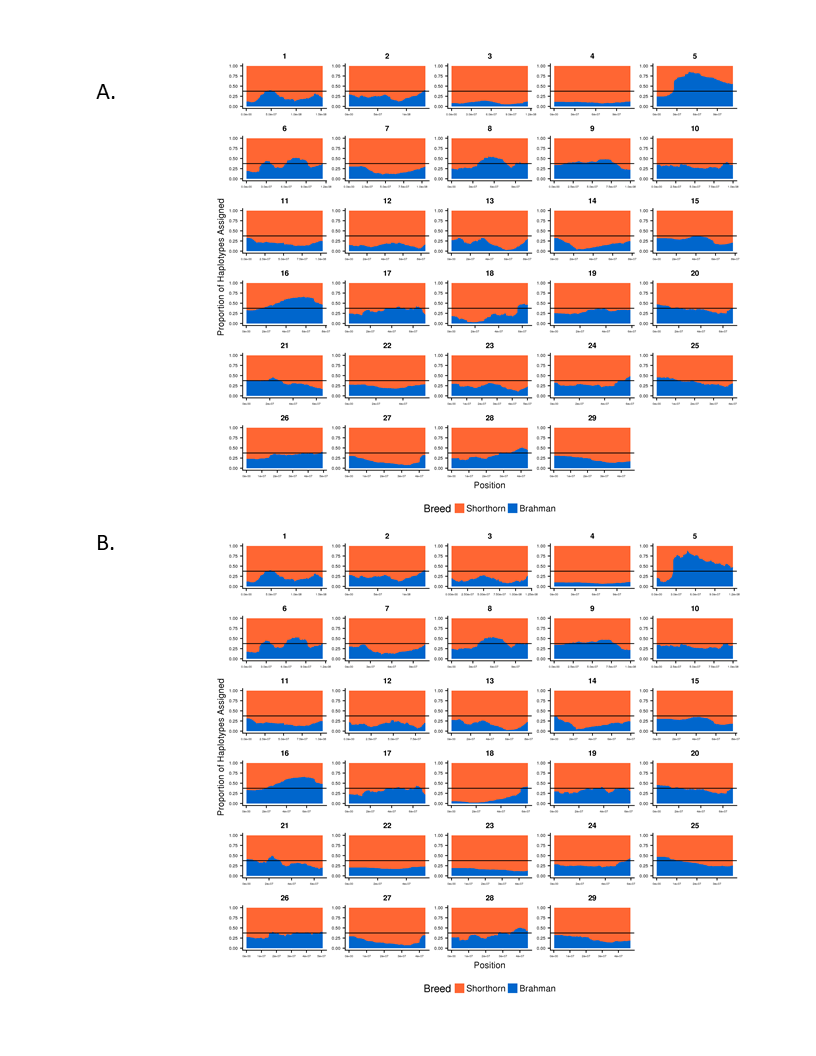
**

**Supplementary Figure S2.** RFMix most likely assignment by chromosome for the: A) CRUMBLER and B) ANCESTRAL reference panels. Brahman (blue) and Shorthorn (orange), for each 100 SNP window for Santa Gertrudis. Horizontal line indicates 3/8 expected Brahman proportion.

**
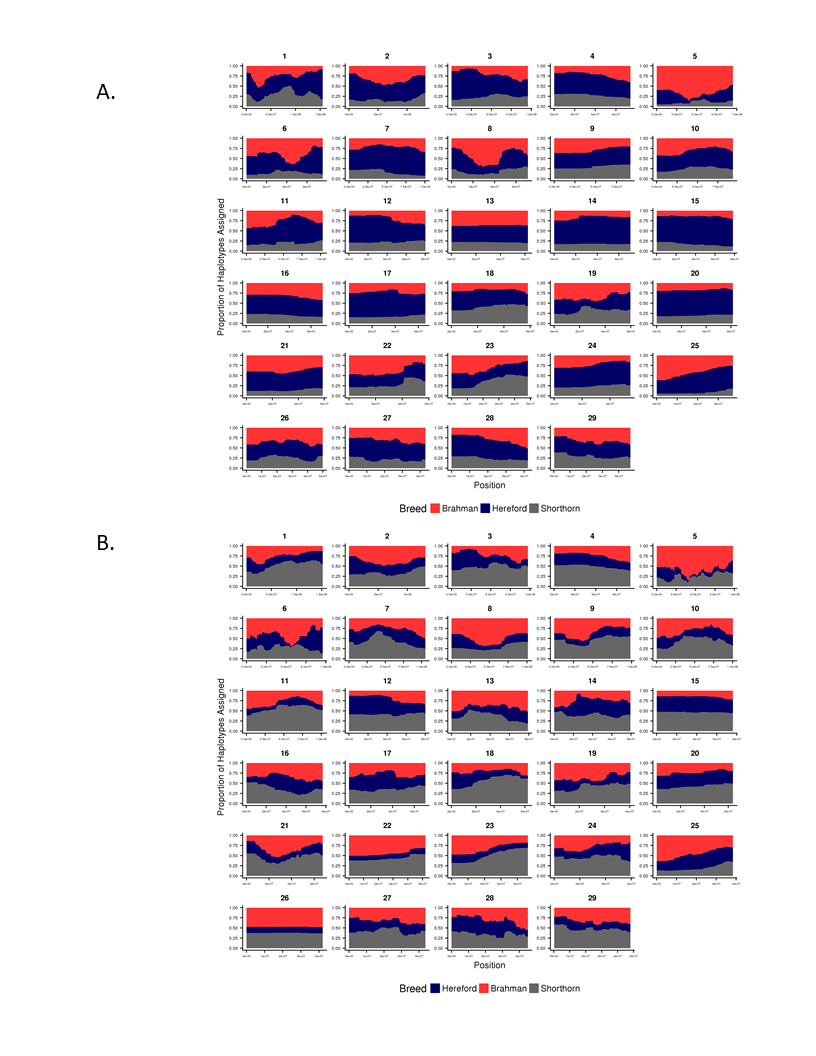
**

**Supplementary Figure S3.** RFMix most likely assignment by chromosome for the: A) CRUMBLER and B) ANCESTRAL reference panels. Brahman (red), Hereford (navy) and Shorthorn (gray), for each 100 SNP window for Beefmaster which is expected to be ½ Brahman, ¼ Hereford and ¼ Shorthorn.


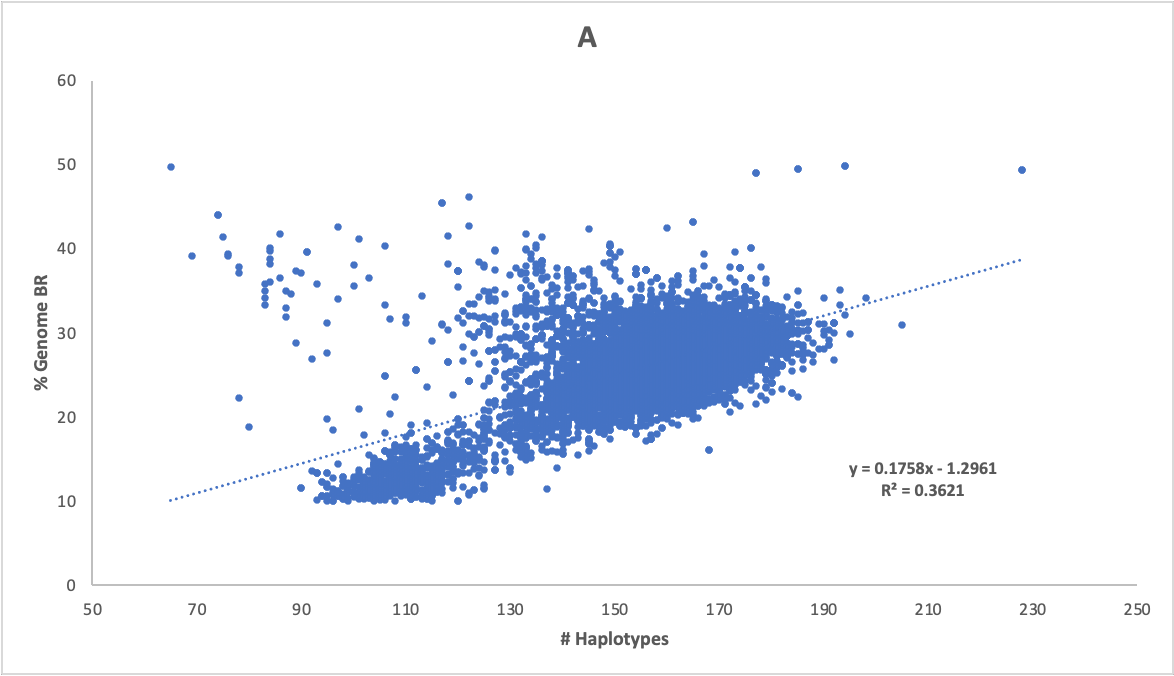


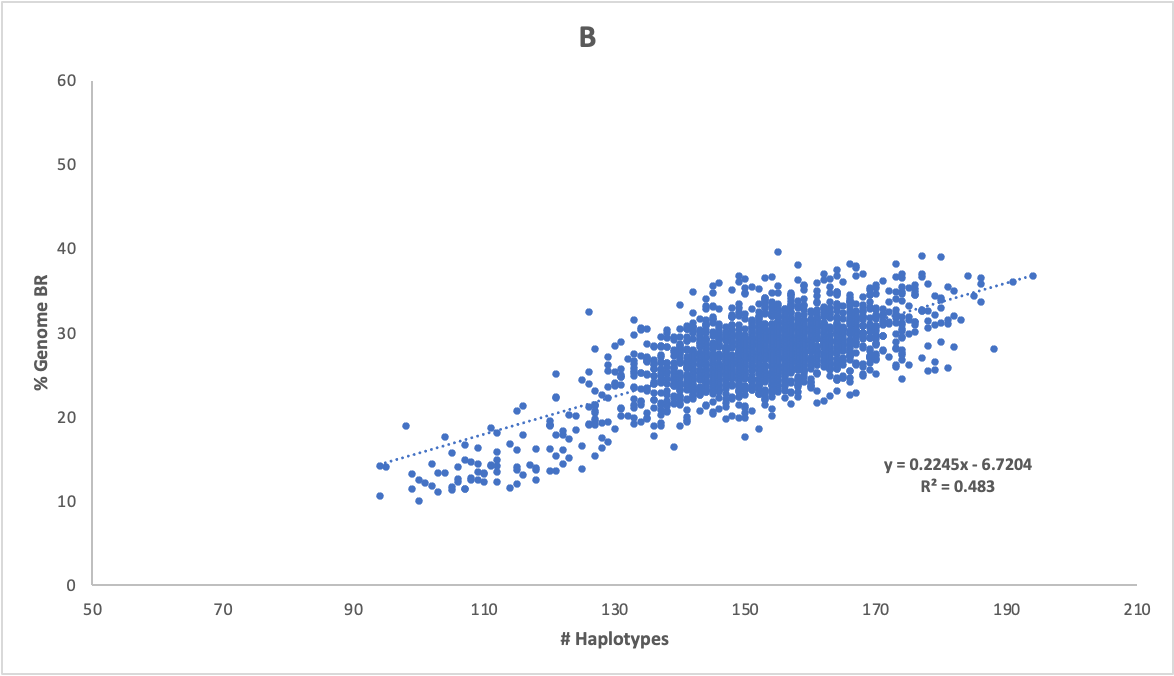


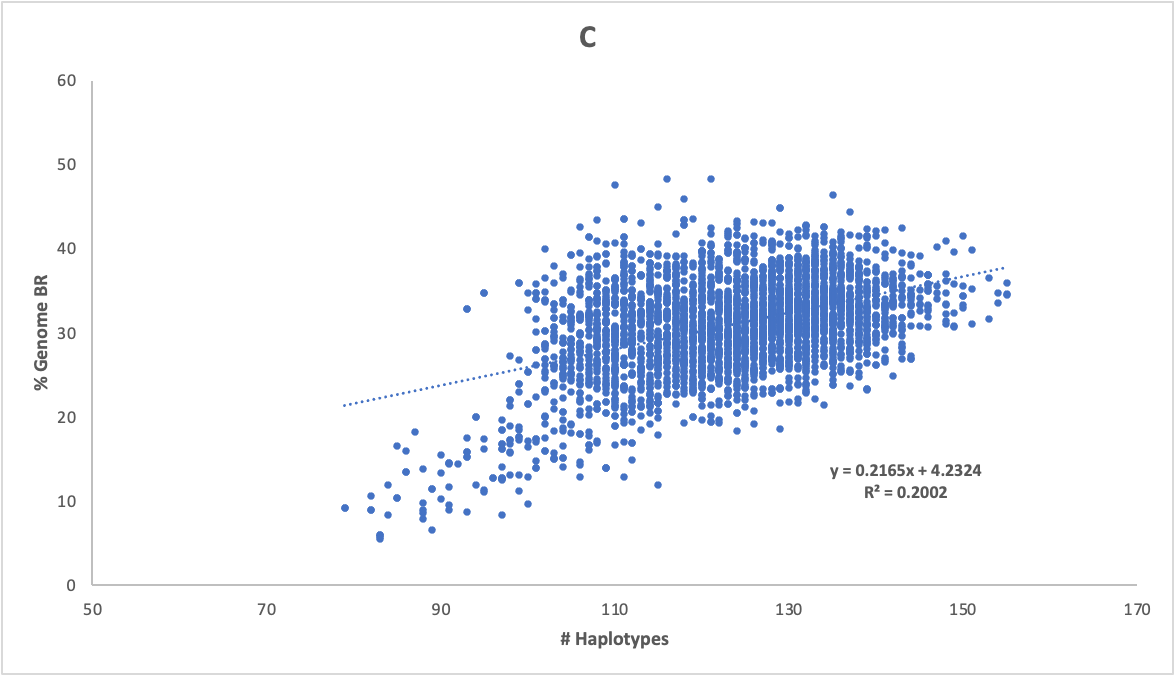


**Supplementary Figure S4.** Relationship between estimated Brahman content and total number of taurine and indicine haplotypes within the diploid genomes of: A) Brangus, B) Santa Gertrudis, and C) Beefmaster animals.


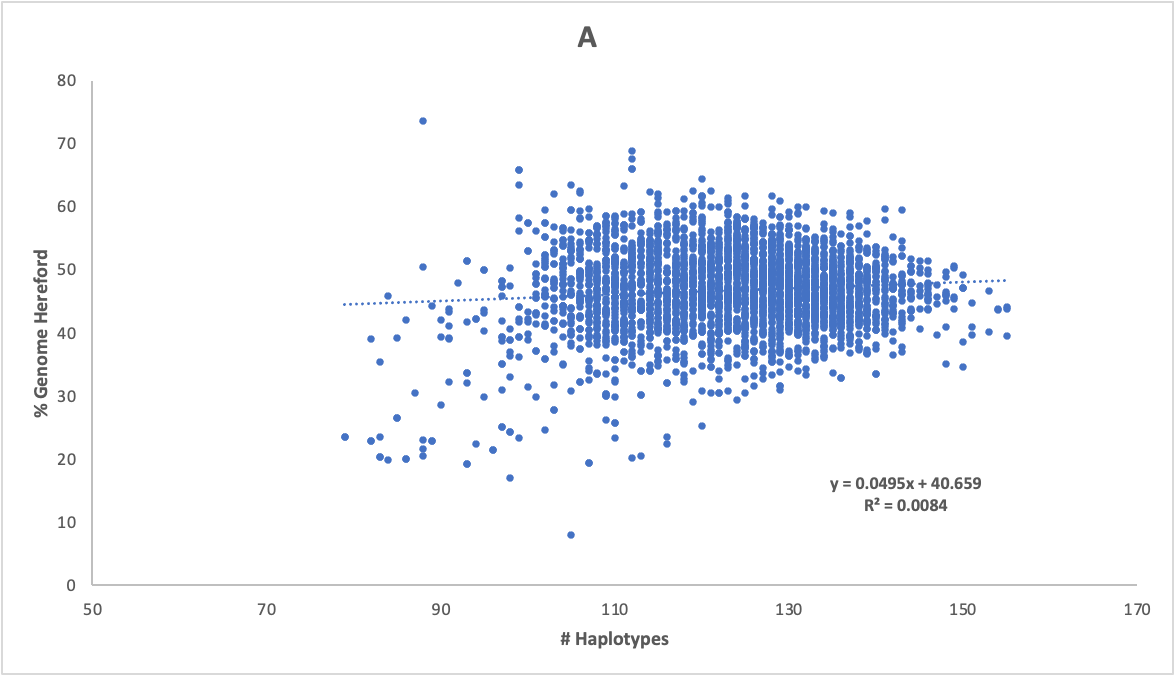


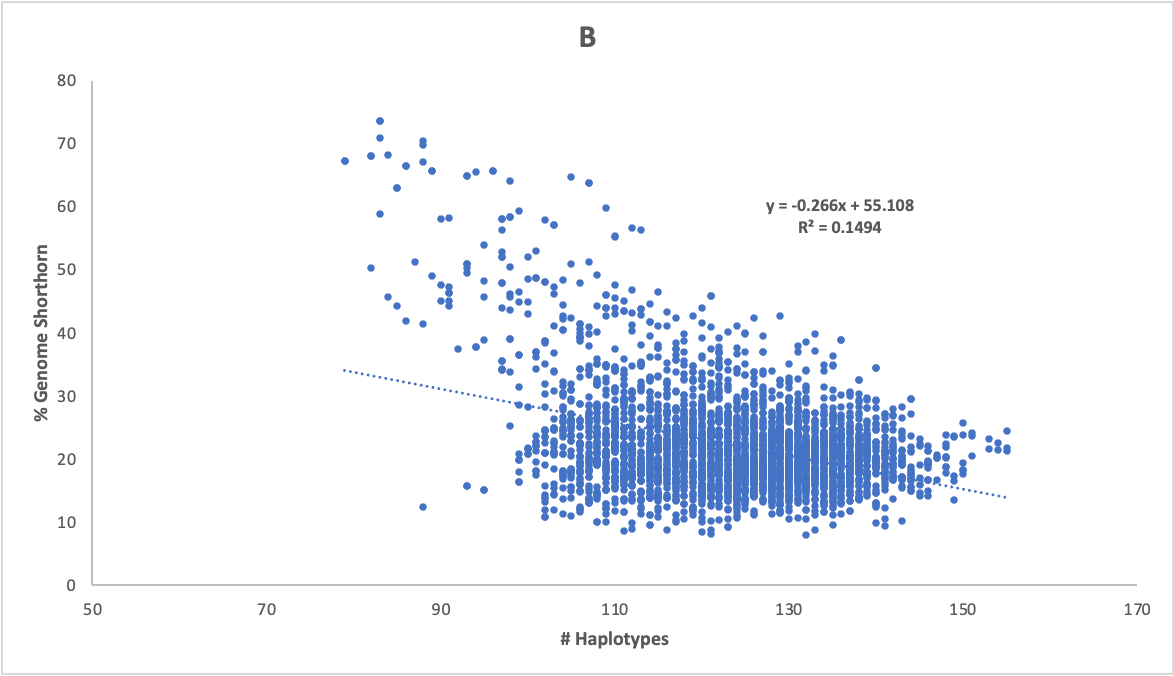


**Supplementary Figure S5.** Relationship between: A) estimated Hereford content and total number of taurine and indicine haplotypes within the diploid genomes of Beefmaster animals, and B) estimated Shorthorn content and total number of taurine and indicine haplotypes within the diploid genomes of Beefmaster animals.

**
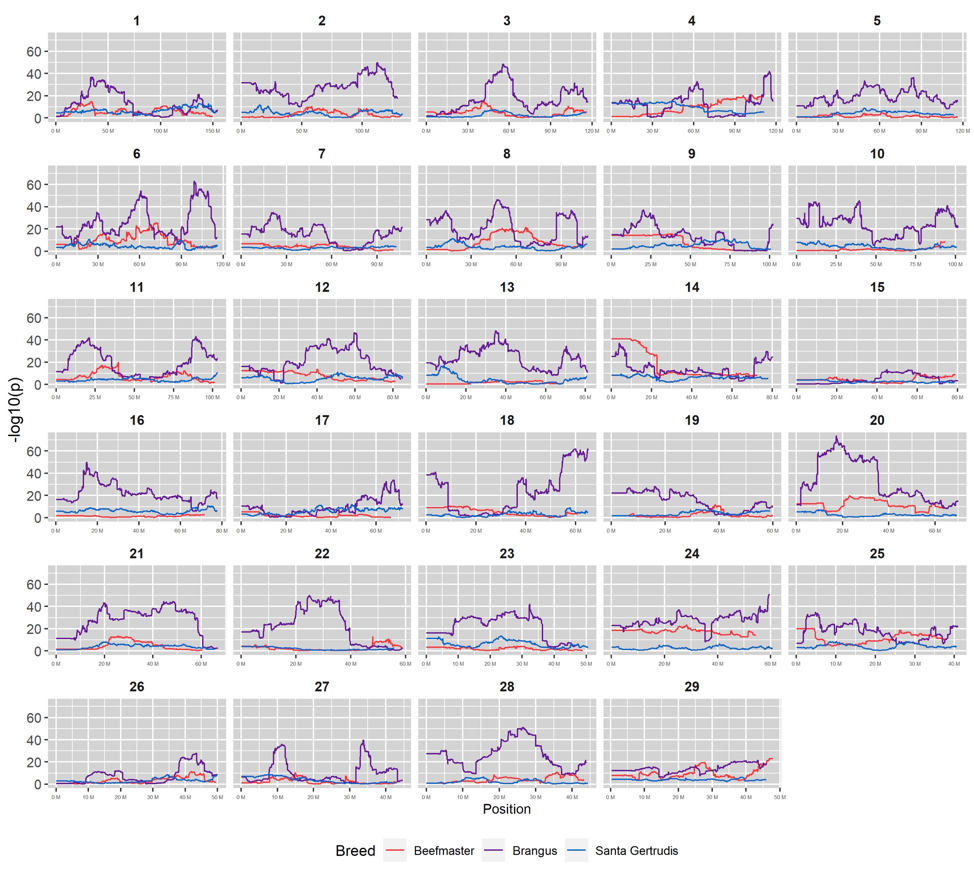
**

**Supplementary Figure S6.** Plots of -log_10_P for tests of each genomic window for differences in Brahman proportion between early- and advanced-generation individuals by chromosome. FDR<0.001 corresponds to -log_10_P from 3.03 to 3.24 in the three American Breeds.

**Supplementary Table S1. Haplotype numbers and lengths per individual by breed of origin in American Breed individuals.**

|  | **Brangus** | | | **Santa Gertrudis** | | | **Beefmaster** | | | |
| --- | --- | --- | --- | --- | --- | --- | --- | --- | --- | --- |
| **Haplotype Metric** | **Brahman**^a^ | **Angus** | **All**^b^ | **Brahman** | **Shorthorn** | **All** | **Brahman** | **Hereford** | **Shorthorn** | **All** |
| Min No. | 22 | 33 | 65 | 24 | 67 | 94 | 13 | 12 | 13 | 79 |
| Max No. | 112 | 116 | 228 | 89 | 107 | 194 | 65 | 65 | 54 | 155 |
| Average No. (SD) | 63.07 11.23 | 90.14 7.91 | 153.21 18.20 | 63.67 8.96 | 88.11 5.57 | 151.77 13.58 | 45.68 7.26 | 49.58 6.49 | 27.90 5.37 | 123.16 11.64 |
| Min Length^c^ (Mb) | 11.891 | 21.353 | 21.460 | 15.313 | 29.707 | 25.433 | 18.016 | 32.439 | 20.617 | 31.817 |
| Max Length^c^ (Mb) | 76.105 | 76.323 | 75.276 | 32.732 | 63.411 | 52.489 | 50.608 | 69.915 | 79.103 | 62.426 |
| Average Length^c^ (Mb) (SD) | 20.005 3.517 | 40.813 5.946 | 32.497 4.821 | 21.240 2.199 | 40.921 4.530 | 32.804 3.358 | 33.430 4.200 | 46.786 5.260 | 39.119 8.598 | 40.4244.091 |

^a^Ancestral breed of haplotype origin.

^b^All haplotypes for all ancestral breeds.

^c^Minimum, maximum and average length of the average breed haplotype length found in each individual.

**Supplementary Table S2. Five most significantly diverged regions for Brahman breed proportion between early- and advanced-generation individuals within each breed.**

| **Chr** | **Start Pos.**^a^ **(bp)** | | **Size**^b^ **(bp)** | ***P_e_*^c^** | ***P_a_*^c^** | **-log10(P)** |
| --- | --- | --- | --- | --- | --- | --- |
|  | | **Brangus** | | | | |
| 6 | 60,208,122 | | 73,238 | 0.16 | 0.43 | 51.46 |
| 6 | 99,180,707 | | 117,847 | 0.15 | 0.44 | 62.88 |
| 18 | 64,905,510 | | 908,860^d^ | 0.22 | 0.56 | 62.48 |
| 20 | 9,415,256 | | 45,774 | 0.25 | 0.59 | 59.03 |
| 20 | 17,251,657 | | 129,190 | 0.30 | 0.72 | 73.54 |
|  | | **Santa Gertrudis** | | | | |
| 1 | 136,564,593 | | 295,678^d^ | 0.16 | 0.39 | 13.01 |
| 2 | 15,167,235 | | 61,570 | 0.12 | 0.33 | 12.00 |
| 4 | 44,394,554 | | 764,346^d^ | 0.03 | 0.22 | 15.02 |
| 13 | 8,978,771 | | 105,103 | 0.16 | 0.43 | 17.38 |
| 23 | 23,306,959 | | 53,495 | 0.17 | 0.41 | 13.67 |
|  | | **Beefmaster** | | | | |
| 6 | 57,575,163 | | 113,193 | 0.42 | 0.67 | 23.11 |
| 6 | 71,493,256 | | 54,556 | 0.44 | 0.70 | 25.59 |
| 14 | 17,128 | | 9,662,604^d^ | 0.09 | 0.37 | 40.95 |
| 24 | 28,241,377 | | 147,362 | 0.13 | 0.34 | 23.47 |
| 29 | 47,085,727 | | 3,504,052^d^ | 0.26 | 0.50 | 23.16 |

^a^Window start coordinate.

^b^Window size in bp.

^c^Brahman proportion within the window within *P_e_* early- and *P_a_* advanced generation animals.

^d^Contiguous 25 SNP windows concatenated by RFMix because haplotype breed assignments were identical.

**Supplementary Table S3. CattleQTLdb queries for QTL with potentially desirable indicine alleles for regions enriched for indicine alleles between early- and advanced-generation American Breed cattle.**

| **Chr** | **Region**^a^ **(bp)** | **Records**^b^ | **Traits**^c^ | **Most Likely QTL**^d^ | **References** |
| --- | --- | --- | --- | --- | --- |
|  |  |  |  | **Brangus** |  |
| 6 | 60,244,741 | 56 | 28 | Calving Ease | Schnabel et al. (2005) |
|  |  |  |  | Interval To 1^st^ Estrus; Fertility Treatments; Non-Return Rate; Sperm Motility; Sperm Abnormalities; Male Fertility; Retained Placenta | Schrooten et al. (2000); Schrooten et al. (2004); Höglund et al. (2009a); Hiltpold et al. (2020) |
|  |  |  |  | Udder Height; Teat Length | Ashwell et al. (2005); Schrooten et al. (2004) |
|  |  |  |  | Weaning Weight; Yearling Weight; Carcass Weight | McClure et al. (2010); Snelling et al. (2010) |
|  |  |  |  | Bovine Respiratory Disease Susceptibility | Neupane et al. (2018) |
| 6 | 99,239631 | 88 | 32 | Calving Ease; Stillbirth | Cole et al. (2011) |
|  |  |  |  | Daughter Pregnancy Rate; Inseminations Per Conception; Insemination Interval | Cole et al. (2011); Höglund et al. (2015) |
|  |  |  |  | Udder Cleft | Cole et al. (2011) |
|  |  |  |  | Chest Girth | Zhang et al. (2017) |
|  |  |  |  | Lean Meat Yield | Doran et al. (2014) |
|  |  |  |  | Mature Body Weight | Crispim et al. (2015) |
|  |  |  |  | Bovine Respiratory Disease Susceptibility; Bovine Tuberculosis Susceptibility; Bovine Viral Diarrhea Virus Susceptibility | Casas et al. (2015); Neupane et al. (2018); Ring et al. (2019) |
| 18 | 69,449,812 | 0 | 0 |  |  |
| 20 | 9,438,143 | 109 | 52 | Birth Weight; Calving Ease; Stillbirth | Seidenspinner et al. (2009); McClure et al. (2010); Cole et al. (2011); Michenet et al. (2016) |
|  |  |  |  | Daughter Pregnancy Rate; Early Embryonic Survival; Fertilization Rate; Sire Conception Rate | Cole et al. (2011); Li et al. (2012) |
|  |  |  |  | Body Depth; Stature; *Longissimus Dorsi* Muscle Area | Cole et al. (2011); Júnior et al. (2016) |
|  |  |  |  | Teat Placement; Udder Attachment; Udder Height; Udder Width; Udder Balance; Udder Depth | Boichard et al. (2003); Schnabel et al. (2005); Cole et al. (2011) |
|  |  |  |  | Average Daily Gain; Maturity Rate; Weaning Weight; Yearling Weight; Mature Weight; Carcass Weight | McClure et al. (2010); Snelling et al. (2010); Doran et al. (2014); Saatchi et al. (2014a); Crispim et al. (2015) |
|  |  |  |  | Infectious Bovine Keratoconjunctivitis Susceptibility; IgG Level; General Disease Susceptibility | Casas and Stone (2006); Casas and Snowder (2008); Maltecca et al. (2008); Garcia et al. (2010); Leach et al. (2010) |
| 20 | 17,316,252 | 41 | 28 | Inseminations Per Conception; Heat Intensity | Höglund et al. (2009a); Galliou et al. (2020) |
|  |  |  |  | Body Depth | Ashwell et al (2005) |
|  |  |  |  | Udder Width | Wu et al. (2013) |
|  |  |  |  | Yearling Weight; Residual Feed Intake | Abo-Ismail et al. (2018); Akanno et al. (2018) |
|  |  |  |  | Heat Tolerance | Howard et al. (2014) |
|  |  |  |  | Trypanotolerance Traits; IgG Level; Tick Resistance | Hanotte et al. (2003); Maltecca et al. (2008); Leach et al. (2012); Sollero et al. (2017) |
|  |  |  |  | **Santa Gertrudis** |  |
| 1 | 138,042,985 | 14 | 10 | Birth Index; Calf Size; Calving Ease; Stillbirth | Sahana et al. (2011) |
|  |  |  |  | Average Daily Gain; Dry Matter Intake; Residual Feed Intake | Peters et al. (2012); Karisa et al. (2013); Abo-Ismail et al. (2018) |
|  |  |  |  | Yearling Weight | Peters et al. (2012) |
| 2 | 15,198,020 | 30 | 22 | Pelvic Area | Michenet et al. (2016) |
|  |  |  |  | Body Depth; Lung Percentage | Ashwell et al. (2005); Morris et al. (2010) |
|  |  |  |  | Teat Placement | Boichard et al. (2003) |
|  |  |  |  | Average Daily Gain; Body Weight Gain; Yearling Weight | Snelling et al. (2010); Lu et al. (2013); Akanno et al. (2018) |
|  |  |  |  | IgG Level | Leach et al. (2012) |
| 4 | 48,216,286 | 26 | 22 | Birth Weight; Calf Size | McClure et al. (2010); Snelling et al. (2010); Höglund et al. (2012) |
|  |  |  |  | Inseminations Per Conception; Conception Rate; Fertility Index; Insemination Interval | Höglund et al. (2014); Liu et al. (2017) |
|  |  |  |  | Udder Depth | Lund et al. (2008) |
|  |  |  |  | Social Behavior | Gutierrez-Gil et al. (2008) |
|  |  |  |  | Yearling Weight | McClure et al. (2010) |
|  |  |  |  | T-Cell Proliferation Following FMDV Peptide Exposure | Leach et al. (2010) |
| 13 | 9,031,323 | 21 | 17 | Inseminations Per Conception; First Service Conception; Heat Intensity | Holmberg and Andersson-Eklund (2006); Galliou et al. (2020) |
|  |  |  |  | Teat Length; Teat Placement; Udder Attachment; Udder Depth | Schrooten et al. (2000); Boichard et al. (2003); Hiendleder et al. (2003); Lund et al. (2008) |
|  |  |  |  | Dry Matter Intake | de Oliveira et al. (2014) |
|  |  |  |  | Weaning Weight; Carcass Weight | McClure et al. (2010) |
|  |  |  |  | Bovine Spongiform Encephalopathy; Bovine Tuberculosis Susceptibility | Zhang et al. (2004); González-Ruiz et al. (2019) |
| 23 | 23,333,707 | 53 | 33 | Stillbirth; Dystocia | Seidenspinner et al. (2009) |
|  |  |  |  | Percent Live Sperm Post-Thaw; Non-Return Rate; Insemination Interval; Scrotal Circumference | Druet et al. (2009); McClure et al. (2010); Höglund et al. (2014); Liu et al. (2017) |
|  |  |  |  | Teat Placement; Udder Depth | Ashwell et al. (2005) |
|  |  |  |  | Dry Matter Intake; Residual Feed Intake | Sherman et al. (2009); Lu et al. (2013); Brunes et al. (2021) |
|  |  |  |  | Weaning Weight | McClure et al. (2010) |
|  |  |  |  | M. Paratuberculosis Susceptibility; Bovine Leukemia Virus Susceptibility; Tick Resistance; Trypanosome Parasite Detection; Veterinary Treatments; White Blood Cell Count; IgG Level | Elo et al. (1999); Hanotte et al. (2003); Leach et al. (2010); Machado et al. (2010); Carignano et al. (2018); Sanchez et al. (2020) |
|  |  |  |  | **Beefmaster** |  |
| 6 | 57,631,760 | 128 | 28 | Calving Ease | Schnabel et al. (2005) |
|  |  |  |  | Non-Return Rate; Estrus Interval; Fertility Treatments; Retained Placenta; Scrotal Circumference | Schrooten et al. (2000); Schrooten et al. (2004); Höglund et al. (2009a); McClure et al. (2010) |
|  |  |  |  | Teat Length; Udder Height | Ashwell et al. (2005); Schrooten et al. (2004) |
|  |  |  |  | Longissimus Area | McClure et al. (2010) |
|  |  |  |  | Growth Rate; Carcass Weight | McClure et al. (2010); Snelling et al. (2010) |
|  |  |  |  | Liver Abscess Susceptibility | Keele et al. (2016) |
| 6 | 71,520,534 | 50 | 21 | Age at Puberty | Hawken et al. (2012) |
|  |  |  |  | Eye Area Pigmentation; Facial Pigmentation; White Spotting | Mészáros et al. (2015); Jivanji et al. (2019) |
|  |  |  |  | Weaning Weight; Yearling Weight; Mature Weight; Carcass Weight; Growth Rate | McClure et al. (2010); Snelling et al. (2010); Crispim et al. (2015) |
| 14 | 4,856,994 | 1711 | 62 | Birth Weight; Calving Ease; Gestation Length; Stillbirth | Kneeland et al. (2004); Kaupe et al. (2007); Maltecca et al. (2008); McClure et al. (2010); Cole et al. (2011) |
|  |  |  |  | Non-Return Rate | Kaupe et al. (2007); Schulman et al. (2008) |
|  |  |  |  | Stature; *Longissimus Dorsi* Muscle Area | Stone et al. (1999); Cole et al. (2011) |
|  |  |  |  | Udder Cleft | Cole et al. (2011) |
|  |  |  |  | Residual Feed Intake | Rolf et al. (2012); Saatchi et al. (2014b); Duarte et al. (2019) |
|  |  |  |  | Average Daily Gain; Weaning Weight; Carcass Weight | Kneeland et al. (2004); Mizoshita et al. (2004); McClure et al. (2010); Espigolan et al. (2015); Michenet et al. (2016) |
|  |  |  |  | Tick Resistance; Bovine Tuberculosis Susceptibility; Gastrointestinal Nematode Burden; IgG Level; Ketosis | Gasparin et al. (2007); Coppieters et al. (2009); Leach et al. (2010); Leach et al. (2012); Richardson et al. (2016); Nayeri et al. (2019) |
| 24 | 28,315,058 | 18 | 14 | Stillbirth | Thomasen et al. (2008); Seidenspinner et al. (2009) |
|  |  |  |  | Fertility Index; Conception Rate; Inseminations Per Conception | Cai et al. (2019); Kiser et al. (2019); Galliou et al. (2020) |
|  |  |  |  | Body Temperature | Dikmen et al. (2013) |
|  |  |  |  | *Longissimus Dorsi* Muscle Area | Peters et al. (2012) |
|  |  |  |  | Feed Conversion Ratio | Sherman et al. (2009) |
|  |  |  |  | Weaning Weight; Carcass Weight | McClure et al. (2010); Michenet et al. (2016) |
|  |  |  |  | IgG Level; Gastrointestinal Nematode Burden; ConA-Induced Cell Proliferation | Coppieters et al. (2009); Leach et al. (2010) |
| 29 | 64,605,989 | 0 | 0 |  |  |

^a^1 Mb region centered on this coordinate.

^b^QTL/Association records returned from CattleQTLdb (September 19, 2021).

^c^Number of traits influenced by QTL/Associations.

^d^QTL for traits known to be selected in U. S. registered beef cattle for which indicine alleles may be desirable.
